# Binocular fusion, suppression and diplopia for blurred edges

**DOI:** 10.1111/opo.12108

**Published:** 2014-01-29

**Authors:** Mark A Georgeson, Stuart A Wallis

**Affiliations:** School of Life & Health Sciences, Aston UniversityBirmingham, UK

**Keywords:** binocular fusion, blur, contrast polarity, diplopia, disparity, edges, interocular suppression

## Abstract

**Purpose:**

(1) To devise a model-based method for estimating the probabilities of binocular fusion, interocular suppression and diplopia from psychophysical judgements, (2) To map out the way fusion, suppression and diplopia vary with binocular disparity and blur of single edges shown to each eye, (3) To compare the binocular interactions found for edges of the same vs opposite contrast polarity.

**Methods:**

Test images were single, horizontal, Gaussian-blurred edges, with blur B = 1–32 min arc, and vertical disparity 0–8.B, shown for 200 ms. In the main experiment, observers reported whether they saw one central edge, one offset edge, or two edges. We argue that the relation between these three response categories and the three perceptual states (fusion, suppression, diplopia) is indirect and likely to be distorted by positional noise and criterion effects, and so we developed a descriptive, probabilistic model to estimate both the perceptual states and the noise/criterion parameters from the data.

**Results:**

(1) Using simulated data, we validated the model-based method by showing that it recovered fairly accurately the disparity ranges for fusion and suppression, (2) The disparity range for fusion (Panum's limit) increased greatly with blur, in line with previous studies. The disparity range for suppression was similar to the fusion limit at large blurs, but two or three times the fusion limit at small blurs. This meant that diplopia was much more prevalent at larger blurs, (3) Diplopia was much more frequent when the two edges had opposite contrast polarity. A formal comparison of models indicated that fusion occurs for same, but not opposite, polarities. Probability of suppression was greater for unequal contrasts, and it was always the lower-contrast edge that was suppressed.

**Conclusions:**

Our model-based data analysis offers a useful tool for probing binocular fusion and suppression psychophysically. The disparity range for fusion increased with edge blur but fell short of complete scale-invariance. The disparity range for suppression also increased with blur but was not close to scale-invariance. Single vision occurs through fusion, but also beyond the fusion range, through suppression. Thus suppression can serve as a mechanism for extending single vision to larger disparities, but mainly for sharper edges where the fusion range is small (5–10 min arc). For large blurs the fusion range is so much larger that no such extension may be needed.

## Introduction

### Binocular fusion

We have two eyes but see one world. Binocular fusion refers to the process, or set of processes, through which information from the two eyes is combined to yield single vision, rather than double vision (diplopia). An obvious benefit of fusion is that (within limits) it renders as single the disparate pairs of images from objects that lie nearer or further than the current binocular fixation distance. Since such objects are normally also perceived in depth as nearer and further, through stereo vision, it is tempting to think that stereo vision and fusion are one and the same process, but that is too simple. Stereo vision and fusion are different and partly dissociable perceptual outcomes of binocular visual processing. It is well known that stereo depth can be seen along with diplopia – at disparities too large for perceptual fusion – and fused single vision from disparate images can occur without stereo depth – in the case of vertical rather than horizontal disparities. Of course, this does not mean stereo and fusion are unrelated processes, but they are dissociable. An early example of this dissociation is Kaufman's letter stereogram^1^ in which depth was seen from disparities in the (coarse) spatial arrangement of letters seen by the two eyes, despite the fact that the letters were different in the two eyes and hence rivalled, rather than fusing, at the level of fine spatial detail.

Parallel processing of binocular information at multiple spatial scales^2^,^3^ is the likely basis for this partial dissociation between fusion and stereo. For spatially broadband images, binocular combination at coarse scales can support stereo vision, while the same disparities processed at finer scales may exceed the fusion limit and result in diplopia.^4^,^5^ This established account relies on the concept of a *size-disparity correlation* – that the disparity range for binocular combination increases with the spatial scale of the stimulus, or with the scale of the underlying mechanism (e.g. receptive field size) that handles the stimulus. Such a correlation was found for the upper and lower disparity limits of stereo vision^6^ and also for the disparity limits of fusion.^5^ We examine it further in our experiments on fusion, but in stereo vision not all studies have revealed it. Smallman & MacLeod^7^ found that contrast sensitivity for stereopsis was best at a spatial frequency that increased progressively as the disparity was made smaller – in agreement with a size-disparity correlation. But Prince & Eagle^8^ found that contrast sensitivity for stereopsis of briefly presented, spatially localized, bandpass Gabor targets did not reveal any simple disparity limit that would imply the existence of a size-disparity correlation in their task, and the upper disparity limit for stereopsis in Gabor targets was about five times larger than for bandpass textures of the same spatial frequency. This extended range of stereo vision is likely to involve contributions from a number of different stereo mechanisms – including coarse, transient or second-order mechanisms.^9^–^13^

### Motivation

Here we re-visit the question of scale dependence for binocular fusion while avoiding the complexities of stereo vision hinted at above. To silence stereo vision we used only vertical disparities, and to strive for simplicity our test stimulus was a single, horizontal, Gaussian-blurred edge shown briefly to each eye. Spatial scale was manipulated by varying the blur of the edge. Blurred edges have a broad, lowpass spatial frequency spectrum, very different from the bandpass targets used in previous studies.^5^,^14^ An advantage in using edges rather than periodic gratings or Gabor patches is that the luminance profile *L*(*x*) for a single edge does not contain gradients of opposite polarity that might rival with each other – and counteract fusion - as disparity increases. The local luminance gradient d*L*/d*x* has the same sign at all locations *x*. Use of step edges may also benefit from a degree of naturalism, in that blurred and sharp edges are abundant in images of natural (and man-made) scenes, and the step edge has a 1/*f* spectrum that is typical of natural images.

We used single edges because of their importance as key features in spatial vision, along with the possibility that they might also be key features in binocular combination. On this theme, Ogle^15^ wrote: ‘It is these contours, the demarcations between light and less light areas, that provide the pattern of the images and the stimuli for fusion when they exist in both eyes’ (p.61). Further, in his classic studies of binocular brightness, Levelt^16^ concluded that the perceived brightness depended on a linear weighted sum of the luminances in the two eyes, but the weight associated with each eye in that sum was determined by the presence and relative strength of contours in the two eyes. The weights add up to 1. This is the ‘law of complementary shares’ (first stated by Hering, but quantified by Levelt), such that an increase in one eye's contribution to the binocular sum entails a reduction in that of the other eye. With a contour in one eye but not the other, the weights can go to 1 and 0 respectively, implying complete monocular dominance. This ‘law’ also applies to binocular summation of suprathreshold contrasts, where the eye with higher contrast also gets a higher weight, and so is disproportionately favoured in binocular combination.^17^,^18^ Such findings and theories on binocular summation sharpen our understanding of the binocular combination of spatial contrast signals, which presumably underlies both stereo vision and fusion.

One goal of our work on fusion is to extend a multiscale model of edge detection^19^ by incorporating binocular combination into the early stages of spatial filtering. The aim here, as a first step, is more empirical – to map out how fusion, interocular suppression and diplopia depend on the disparity, spatial scale (blur), and relative contrast of the edges in each eye. To do this, we need to develop an effective method for assessing whether fusion, suppression of one edge, or diplopia have occurred. Our experiments were motivated by the work of Schor *et al*.^5^ but with some significant differences in method. Schor *et al*. used high contrast, bandpass (difference-of-Gaussian) targets, while we used single, blurred edges of moderate contrast. Schor *et al*. used prolonged presentation, with the method of adjustment and a rather complex perceptual decision: ‘Disparity was increased by method of adjustment until the subject reported a slight doubling, an increase in width or a lateral displacement of the DOG pattern. A lateral displacement would indicate suppression of one image’ (p.662). We simplified the observer's task by using discrete trials with brief presentations, along with a categorical judgement (a button-pressing response), thus aiming to evaluate the *probability* of fusion, suppression and diplopia, and the way these varied with disparity.

In experiment 1, the goal was to distinguish between fusion and not-fusion, and so observers reported whether they saw a single central edge, or not. It became clear in pilot sessions, however, that the ‘not central’ category included both diplopic edges and single edges that were not central, and it seemed likely that the latter might reflect suppression of one of the two edges. Hence in experiment 2 all three response categories were used. We reasoned (like Ono, Angus & Gregor^20^) that with fusion the perceived target position should be central, with suppression of one image it should appear offset (by half the disparity), and with diplopia (by definition) the observer should see two edges rather than one. A potentially serious complication, however, is that in the face of blur and positional noise a fused edge might appear offset, or an offset edge might appear central. In addition, the probability of those judgements would depend on the observer's positional criterion for reporting ‘central’ vs ‘offset’. Thus the relation between behavioural judgements and perceptual states is not straightforward. To resolve this problem we developed, and validated through simulation, a descriptive model that was fitted to the data and allowed us to disentangle the perceptual states (fusion, suppression, diplopia) from the perturbations imposed by positional criterion and noise factors.

## Methods

A single-interval method of constant stimuli was used to explore fusion, diplopia and suppression of luminance edges, across a range of vertical disparities, spatial scales and (for experiment 3) levels of contrast imbalance between the eyes.

**Figure 1:**
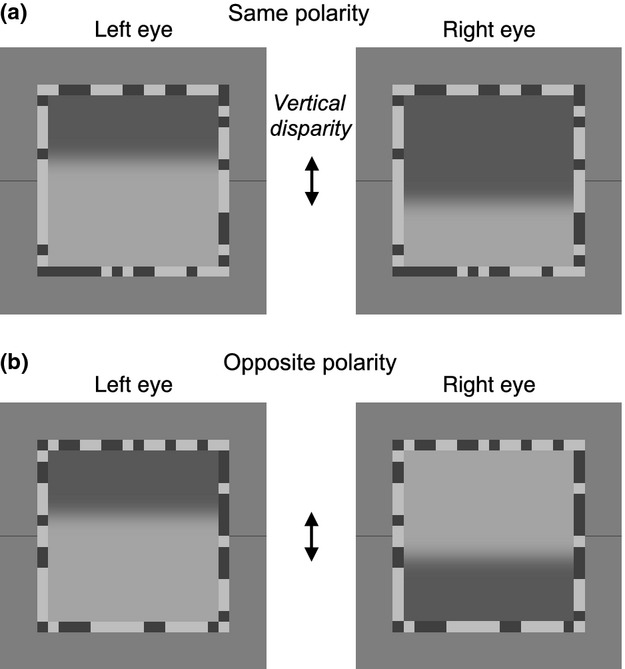
Image pairs used in the experiments. The test image in each eye was a single, horizontal, Gaussian-blurred edge, while the textured border served as a ‘fusion lock’ to stabilise binocular convergence. Thin lines marked the (vertical) centre position as a reference for ‘central’ vs ‘offset’ judgements. (a) Same test edge polarity (experiments 1–3). (b) Opposite edge polarity (experiment 3 only). Test edge blurs B ranged from 1 to 32 min arc, and for each blur the entire image was scaled in size accordingly. Test image geometry was therefore scale-invariant. Vertical disparity between left and right edge positions (arrowed) ranged from 0 to 8.B.

The test image for each eye (*Figure 1*) was a horizontal Gaussian-blurred edge of blur B = 1, 2, 4, 8, 16 or 32 min arc. Image width and height were scaled with edge blur, and ranged from 20 to 640 min arc (16–512 pixels) at the viewing distance of 107 cm.

Image arrays were generated in *Matlab* on a Macintosh G4 computer and displayed using *PsychToolbox* software^21^ on a Clinton Monoray monitor with a fast-decay yellow-green phosphor, calibrated and gamma-corrected using a Minolta LS110 computer-controlled digital photometer. A Cambridge Research Systems Bits++ box was used in Bits mode (experiments 1 and 2) or Mono++ mode (experiment 3) to render pseudo-14-bit (Bits mode) or actual 14-bit (Mono mode) greyscale resolution. Images were viewed through frame-interleaving FE1 goggles (CRS Ltd., www.crsltd.com) to present separate images alternately to the two eyes with minimal crosstalk.^22^ The high frame rate (150 Hz; 75 Hz per eye) ensured that the alternating display appeared as a steady image with no visible flicker.

The horizontal test edge was presented dichoptically with a vertical disparity *d* (in units of blur, B) from 0 to 8B. Disparity was induced by shifting the edge upwards in one eye, and downwards in the other eye by *d*/2. The Michelson contrast of the test edge was 0.3 (except in experiment 3 where left/right contrast ratio varied but geometric mean edge contrast was 0.3). There were two polarities for edge contrast (light above; dark above) and two signs of vertical disparity (right eye higher; left eye higher).

The test image was surrounded by a binary noise ‘vergence lock’ border (*Figure 1*), of width B, texel size B and contrast 0.5. Flanking, dark horizontal lines marked the vertical position of the centre of the image. These lines were the same luminance as the dark pixels in the noise border. They were two pixels wide and their length (4–112 pixels) was proportional to B for all images except the largest (B = 32 min arc), where it was truncated from 128 pixels to 112 pixels by the edge of the screen. The background was a full-screen at mid-luminance (25 cd m^−2^ as seen through the goggles).

Each session consisted of five repetitions (experiments 1 and 2) or 1 repetition (experiment 3) of all of the conditions for a single blur, in randomized order. On each trial, the pair of test edges was presented for 200 ms with the noise border and flanking lines, after which the test edges were replaced by a blank (zero contrast), mid-grey image. The observer was instructed to fixate the centre of the image, and had unlimited time to make a response by pressing one of two buttons (experiment 1), one of three buttons (experiment 2), or one of five buttons (experiment 3). The nature of the choice is described below for each experiment. These were perceptual not forced-choice performance tasks, and so no feedback was given. The noise border and flanking lines were displayed continuously throughout the experiment, and the noise border was refreshed at the end of each trial by random selection from a set of 50 such borders.

Observers completed six (experiments 1 and 2) or 12 (experiment 3) sessions, but the first session for each blur was discarded as practice. The image blur used in each session was randomly selected, with the constraint that each blur was used equally often. Data were pooled over 20 repetitions of the four sub-conditions (two signs of contrast and two signs of disparity) giving a total of *N* = 80 trials per disparity and blur for each observer.

There were four observers, aged 20–46. One (SAW) was aware of the purpose of the experiment, but the other three were not. All had experience of psychophysical experiments (though ASB and SGB had less experience than SAW and DHB), and all had normal or corrected-to-normal acuity and normal binocular vision. Stereo acuity was checked before the experiment and found to be within normal limits. Each gave informed consent.

### Experiment 1

In addition to the dichoptic conditions described above, experiment 1 included monoptic conditions, where both test edges were presented to the same eye, combined by linear summation of their contrast profiles, giving a total contrast of 0.6, but no change of mean luminance. The other eye saw a blank image at mid-grey luminance, but with the usual noise border and flanking lines. These monoptic (left or right eye) conditions were randomly interleaved with the dichoptic conditions in each session.

The two-choice task: Observers were instructed “Respond ‘1’ if you see a single central edge or respond ‘2’ if you see two edges or a single edge displaced from the centre.” To reduce the impact of finger errors, observers had the option to alter the response just given, by pressing an ‘error button’. This switched the response to the opposite category.

### Experiment 2

The three-choice task: Observers had three response categories: ‘single central edge’, ‘single offset edge’ or ‘two edges’. The ‘error button’ now vetoed the most recent response, and a second key press denoted the observer's corrected response. There were no monoptic conditions. In all other respects, the method matched experiment 1.

### Experiment 3

In experiment 3, extra conditions were added where the test edge contrast was of opposite sign in the two eyes, and the test edge contrasts could be equal or unequal in the two eyes to allow a broader examination of interocular suppression. From a base contrast of 0.3, contrast imbalance was produced by increasing the test edge contrast in one eye by 0, 3 or 6 dB and decreasing it in the other eye by the same amount. Thus the left:right contrast ratios were −12, −6, 0, 6, 12 dB (i.e. ¼, ½, 1, 2, 4). At 0 dB, contrasts were equal (0.3) in each eye. These extra conditions greatly increased the size of the experiment, so only one edge blur (B = 8 min arc) was used.

There were 680 conditions. Of these, 360 were derived from five contrast ratios, two pairings of contrast sign (same or opposite in the two eyes), two signs of contrast, two signs of disparity and nine disparities (0–8B). The other 320 were zero disparity conditions, designed to examine the observers’ positional noise (which limits their ability to discriminate a central edge from a non-central edge) and to determine the placement of their criteria. Here, a binocular, zero-disparity edge was displaced upwards or downwards from the centre by one of eight offset magnitudes (0.25–2B in 0.25B increments). These 320 conditions were derived from five levels of contrast imbalance, two pairings of contrast sign, two signs of contrast, two directions of offset (up or down) and eight offset magnitudes.

The five-choice task: Response categories were: ‘2 edges’, ‘1 central edge, light at top’, ‘1 central edge, light at bottom’, ‘1 edge above centre’ and ‘1 edge below centre’. The ‘error button’ was not used. One session consisted of a single presentation of all 680 conditions, plus a second trial for all the 0 dB conditions, in random order (816 trials in total), taking about 35 min. Each observer completed 11 sessions, but the first was discarded as practice. Observer SGB was no longer available and was replaced with a psychophysically naïve observer (RH), who was unaware of the purpose of the experiment and had normal binocular vision. For the present paper, the five-choice data were collapsed to the three categories of experiment 2 to enable the same kind of analysis and modelling, described next.

## Modelling

### Modelling the data to derive the characteristics of fusion and suppression

**Figure 2:**
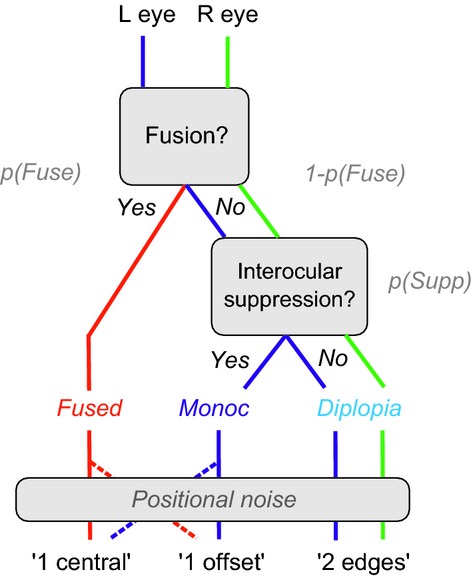
Functional relations between binocular fusion, interocular suppression and diplopia, represented as a decision tree. With added noise, this schematic structure leads to our model for interpreting the experimental data. *Fused*, *monoc* and *diplopia* are mutually exclusive perceptual states. These states map onto decisions via positional noise and a decision criterion that need to be accounted for when interpreting the behavioural responses.

We begin with a general scheme for thinking about fused and non-fused vision (*Figure 2*). For a given external feature presented to both eyes, we assume that there are two mutually exclusive internal states – fusion and non-fusion. *Fusion*, by definition, must arise from some mechanism that combines signals from both eyes, while non-fusion arises from monocularly-driven mechanisms that do not combine. Non-fused percepts, one from each eye, can co-exist (double vision) or one of them may be largely or completely *suppressed*. On the other hand, it appears that fused and non-fused percepts (from a given pair of input features) never co-exist.^14^ This could imply (1) a second process of ‘fusional suppression’^23^ in which the fused representation suppresses the non-fused ones, or (2) that monocular signals are routed to perception only through binocular pathways, so when monocular signals are fused, their separate identity is lost. Either way, when fusion occurs the non-fused signals are not available and so ‘triplopia’ – seeing fused and diplopic images together – is never experienced.^14^ Our descriptive model simply assumes that, whatever the underlying mechanism might be, fusion and non-fusion are mutually exclusive. Blake & Boothroyd^24^ found evidence for a closely related idea – that fusion takes precedence over rivalry. When vertical gratings in both eyes were fused, they ceased to rival with a horizontal grating that was shown only to one eye.

**Figure 3:**
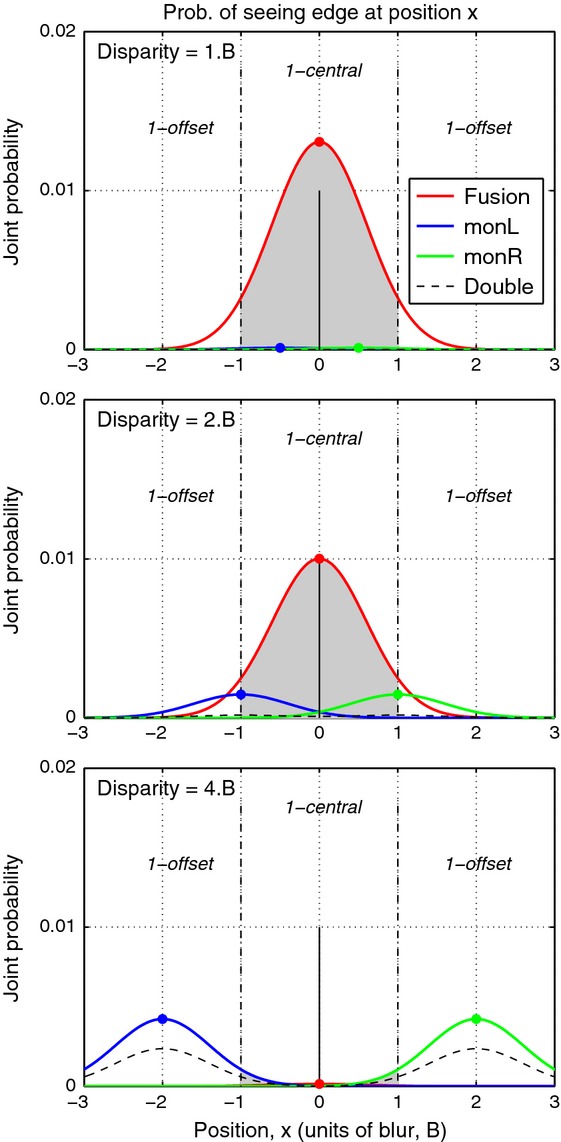
From perceptual states to behavioural responses in the three-choice task. Red curves show for a given disparity the joint probability that an edge will be fused and seen at position *x*. Total area under the red curve is *p*(*Fuse*) – the probability of fusion for a given disparity (top to bottom: disparity = B, 2B, 4B). Shaded grey area under the fusion curve shows the proportion of fused trials that fall within the criteria (vertical broken lines) hence judged as ‘1 central’. Top panel: The proportion of 1-central responses from fusion under-estimates the probability of fusion by an amount that depends on the criterion setting and the noise level. Remaining trials (white areas under the red curve) are ‘false alarms’: fused, but judged as offset. Middle: at a larger disparity *p*(*Fuse*) is reduced, and *monoc* states (split between left eye, blue; right eye, green) increase. A proportion of those trials (shaded grey under the blue & green curves) will be judged as ‘1-central’ rather than ‘1-offset’. Bottom: at an even larger disparity fusion is absent, and responses are mainly split between ‘1-offset’ and ‘double’ (dashed curve). [Model parameters for this illustration were: Fusion range σ_*f*_ = 2.5B, suppression range σ_*s*_ = 5.B, noise S.D. *n *=* *0.6B, criterion placement *c* = ±1, *q *=* *4.]

When the fused state prevails, the model observer reports a single feature. Mean position of the fused feature is modelled here as a contrast-weighted average of the monocular positions, so when left and right eye contrasts are equal, mean position *x*_0_ should be central (*x*_0_* *=* *0). But in the face of positional noise, the encoded position may be offset from the centre, and if that noise-induced offset exceeds a criterion amount, the observer will report ‘1 offset’ rather than ‘1 central’ (see *Figure 3*, top panel). Fusion holds over a limited range of disparities (Panum's limit), so we suppose that the probability of fusion *p*(*Fuse*) falls smoothly with increasing disparity *d*. If on a given trial there is no fusion and no suppression then both monocular edges survive, the outcome state is *diplopia* and the observer reports ‘2 edges’.

But if the fused state does not prevail, *and* suppression of one monocular feature occurs, the outcome is denoted *monoc*, and the observer reports a single edge. We assume, perhaps counter-intuitively, that the probability *p*(*Supp*) of suppression of one monocular feature (but not both) is greatest at zero disparity, and falls with increasing disparity. We shall see later that this general assumption makes good sense of the data. It does not imply that monocular features are often seen at small disparities, however, because a single monocular edge is seen only in the non-fused state, and at small disparities the non-fused state has a low probability.

The mean position of a *monoc* edge corresponds to its monocular position (±*d*/2), but depending on the criterion *c* for reporting ‘offset’, and the amount of positional noise *n*, a single *monoc* edge may be reported as ‘central’ if its position *x* falls within the range –c ≤ x ≤ c, or ‘offset’ if |*x*| > *c* (see *Figure 3*, middle panel).

In short, the behavioural responses about perceived position (‘offset’ or ‘central’) do not uniquely identify the states (*monoc* or *fusion*) that gave rise to them, and the extent of this uncertainty (noise) is unknown. But the variation of the positional judgements over disparity may still carry useful information about fusion and suppression, and it may be possible to estimate the noise through modelling. This key idea is tested (and confirmed) below in a simulation. In modelling experimental data our goal is to allow the internal probabilities *p*(*Fuse*) and *p*(*Supp*) to be teased apart, and estimated as functions of disparity, from the behavioural data.

### Model equations

We formalize the above proposals as follows. The probability of suppression falls as a Gaussian function of disparity, with a spread constant σ_*s*_:

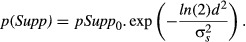
(1)

With this formulation σ_*s*_ is the half-width at half-height (hwhh) – the disparity at which *p*(*Supp*) falls to *pSupp_0_*/2 – and it defines the disparity range for suppression. For *matched* monocular features (i.e. the same polarity, orientation, contrast and blur, as in experiments 1 and 2), we assume *pSupp_0_ *=* *1, but we consider later the idea that *pSupp_0_* ≤1 for edges of opposite polarity. Similarly, for fusion:

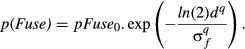
(2)

As before, σ_*f*_ is the disparity range for fusion, and we propose that for matched features *pFuse_0_ *=* *1; at zero disparity fusion always prevails. But for opposite polarities there might be no fusion at all: *pFuse_0_ *=* *0. Note also that this function is a *generalised Gaussian* (where the steepness parameter *q* replaces the usual 2). When *q *>* *2, the function has a flatter top and steeper sides than the Gaussian, and when *q* is very large the function becomes a square box of width 2σ_*f*_.

If fusion does not occur then the outcome may be *diplopia*, but if one monocular edge is suppressed the outcome is *monoc*, with a probability:

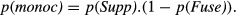
(3)

We denote the three kinds of behavioural response as ‘1 central’, ‘1 offset’ and ‘2 edges’. The diplopia decision (‘2 edges’) appears not to involve any positional judgement, and so for simplicity we assume that the decision between one and two edges is unaffected by positional noise, hence

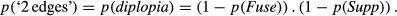
(4)

The two other response categories are positional judgements, whose probabilities have several components. Given a *monoc* edge whose mean position is *x*_1_ = *d*/2, or a *fused* edge whose mean position is *x*_0_, with trial-to-trial standard deviation *n*, we compute the (two-tailed) conditional probability that the edge falls outside the criterion boundaries set at *x = ±c*:

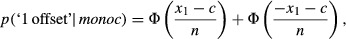


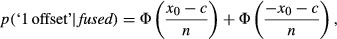

where Φ is the standard normal integral. These are then combined to give the simple probability:

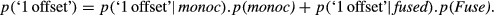
(5)

Similarly, for ‘1 central’ responses we have conditional probabilities:

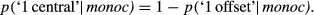


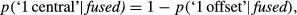

leading to:

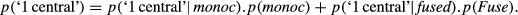
(6)

In summary, the model describes the way fusion and suppression vary with disparity via the equations for *p*(*Fuse*) and *p*(*Supp*)*,* and translates those functions into behavioural response probabilities by allowing for both positional noise and the decision criterion that divides ‘central’ from ‘offset’ judgements. There are five free parameters to be estimated (σ_*f*_*,* σ_*s*_*, q, c, n*). The first two – fusion and suppression ranges – are of greater interest because, if estimated accurately, they describe the disparity dependence of two key processes in binocular vision.

### Model fitting

#### Experiment 2

The descriptive model (above) was fitted to datasets from experiment 2 comprising 27 response probabilities (nine disparities × three response types), separately for each observer and each blur, using the method of maximum likelihood. Goodness-of-fit was assessed (1) by computing the deviance and comparing it with the chi-square distribution for 18 d.f. [for these multinomial data, d.f.* = m*.(*r*−1), where *m* is the number of conditions (disparities) and *r *=* *3 is the number of response alternatives] and (2) by routinely plotting the deviance residuals (analogous to the residual sums of squares, RSS) as a function of disparity and examining these for systematic structure that would indicate a poor fit.

We first allowed all five parameters to be adjusted (by *fminsearch* in *Matlab*) to fit each dataset (denoted as “5-free”). After extensive exploration, we chose to prevent unstable combinations of parameters, by constraining *q* to the range 2 ≤ q ≤ 5, σ_*s*_ ≥ 0.5 and σ_*f*_ ≥ 1. Fits were generally good, but to reduce the danger of over-fitting, and to remove some instability caused by a very evident trade-off in the effects of the *c* and *n* parameters, we also fitted with four free parameters and a fixed criterion (*c *=* *1), denoted “4-free, *c *=* *1”. It was plain that the *c/n* ratio was more important than the absolute values. We chose *c *=* *1 because it was close to the mean obtained with 5-free. In similar vein we also fitted with 3-free, *c *=* *1, *q *=* *4.

#### Experiment 1

The two-choice format of experiment 1 did not deliver the three response categories needed to fit the model, but to allow some comparison with experiment 2 we again used the generalised Gaussian function to derive an empirical description of the disparity range σ_1_ for ‘single-central’ responses. The steepness *q* and peak probability *A* were fitted parameters (2 ≤ q ≤ 10, 0 ≤ *A* ≤ 1):

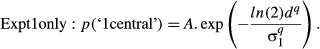


#### Experiment 3

Fitting was done separately for each observer and each contrast ratio, but data for same and opposite polarities were fitted together, as follows. The experiment yielded direct experimental estimates of noise and criterion for each observer, and these were used as fixed parameters *n, c*. As before, *pSupp_0_* was set to 1 for the same polarity conditions. That left four parameters to be fitted: fusional range, suppression range (same polarity), suppression range (opposite polarity), and peak probability of suppression *pSupp_0_* for opposite polarity.

### Testing the validity of the model

Our experiments did not ask observers to distinguish directly between fusion and non-fusion, because we suppose that if the observer sees a single edge he or she has either a weak or no basis for knowing whether this edge was mediated by monocular or binocular mechanisms. But it is safe to assume that observers can make judgements about the position of an edge, and at least plausible that these position judgements would differ systematically for *fused* and *monoc* edges. Hence by modelling these judgements we aim to recover information about the underlying probabilities of fusion and suppression across disparity – but we cannot know *a priori* that this is possible. Having laid out a basic, perhaps uncontroversial, set of assumptions in the model above, we have seen that with positional noise the *fused* and *monoc* states contribute to *both* the 1-central and 1-offset response categories (*Figures 2, 3* and Equations 5 and 6). Perhaps the behavioural responses are too confounded to be useful? The issue is one of content validity: how do we know that the parameters returned by fits to the data (especially σ_*f*_*,* σ_*s*_) actually reflect processes or states of fusion and suppression in the observer's visual system? We addressed this with a simulation.

**Figure 4:**
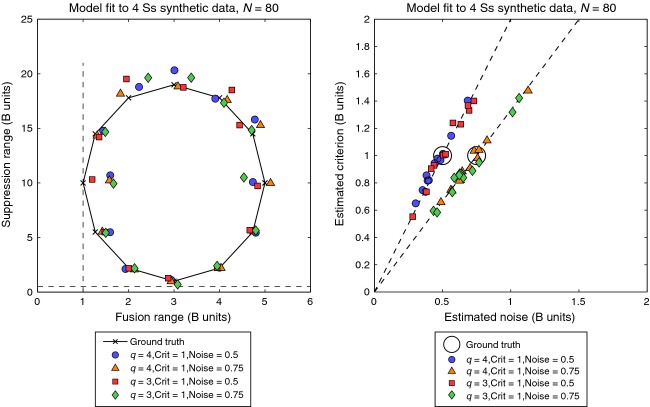
Results of a simulation to test the descriptive model's ability to recover accurate estimates of fusion range and suppression range. Twelve pairs of ‘true’ values (marked by x's) were used to generate synthetic data (*N* = 80 trials, four simulated observers, as in our experiments), to which the model was then fitted with five free parameters. Coloured symbols (left panel) show that for various combinations of the three other generative parameters, the model produced reasonably accurate mean estimates of fusion range and suppression range – i.e. the symbols fall close to the x's. This was true despite strong redundancy (correlation) in the effects of two other parameters. Right panel shows that absolute values of the noise and position criteria (large circles) were not so well recovered. Within limits, it was the ratio of noise to criterion that mattered, and this was accurately estimated (dashed lines).

For 48 different combinations of parameters we used the model to generate synthetic data in the same format as experiment 2, i.e. *N* = 80 trials for each of nine disparities and four (simulated) observers. The simulated datasets produced a range of patterns of response variation quite similar to those observed experimentally. The model was then fitted to the synthetic data, in the same way as the real data (“5-free”). *Figure 4* (left) plots the estimated pairings (σ_*f*_*,* σ_*s*_) as coloured symbols to be compared with ‘ground truth’ (x's lying on a circular locus). It is clear that the model tracked the ‘true’ values fairly closely around all parts of the parameter space. This contrasts with *Figure 4* (right) where values of criterion *c* and noise *n* were not so reliably estimated, but their ratio *c/n* was.

We conclude from this simulation that the model can in principle recover values of the disparity ranges (σ_*f*_*,* σ_*s*_) that are close to the true values, even though two other parameters (*c,n*) were confounded in the 5-free fit. This gives us confidence that our experimental estimates of the disparity ranges (σ_*f*_*,* σ_*s*_) are meaningful and valid. [The interested reader is directed to Appendix B, where we describe another way of estimating the parameters, using AIC and multi-model inference to avoid the confound between *c* and *n*. The resulting mean estimates were very similar to those produced by the three more conventional methods (Appendix A, Table A1).]

## Results

### Experiment 1

**Figure 5:**
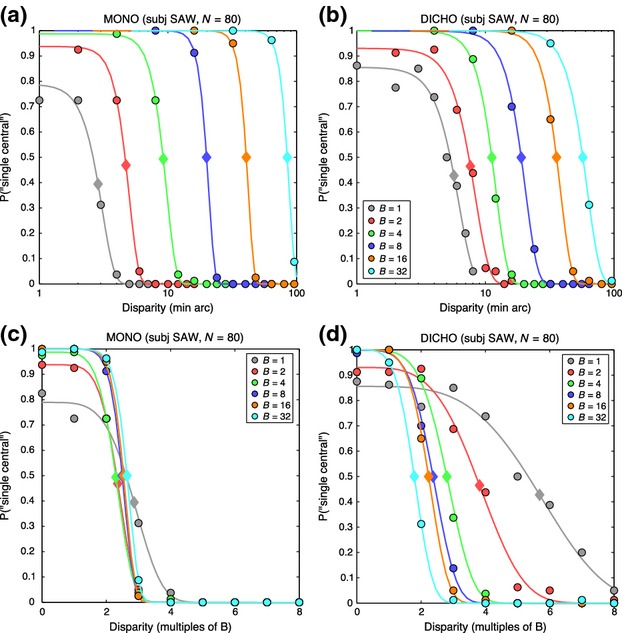
Experiment 1. Data from the 2-choice procedure for one observer (SAW). (a) Monoptic condition (both edges in the same eye). Symbols show the proportion of trials on which the observer reported a single, central edge, as a function of absolute disparity (in min arc, log scale). Different colours represent different edge blurs, in min arc, as shown. Smooth curves are the fits of a generalised Gaussian function (see text). Diamond symbols on the curves lie at half the peak height of each fitted curve, estimating the disparity range for single-central responses. (b) Similar to (a), for dichoptic edges. The absolute disparity range increased markedly with the scale (blur) of the edge. (c, d) Same data as (a, b), but with disparity re-plotted as a multiple of the blur B. Scale invariance was more evident for the monoptic task (c) than the dichoptic (d).

For the two-choice task, equivalent to a ‘yes-no’ task, it is sufficient to plot the proportion of ‘1 central’ responses as a function of disparity. *Figure 5* illustrates data for one of the four observers. Fitted curves are the generalised Gaussian function defined above, and they are usefully summarised by their disparity ranges σ_1_, at which responses fell to half their peak rate, marked by diamond symbols. Clearly, for both monoptic and dichoptic conditions (*Figure 5a,b*), the disparity range increased markedly with the scale (blur) of the edge. The degree of scale invariance in these data can be visualised by expressing disparity as a multiple of the edge blur B (*Figure 5c,d*). For example, at B = 8 min arc, a disparity of 16 min arc converts to a disparity of 2B. For this observer (and indeed all four observers), the monoptic range was scale-invariant – i.e. almost constant with a mean of about 2.5B (means over the six blurs: SAW 2.51, DHB 2.45, ASB 1.76, SGB 3.03). For dichoptic viewing, the range was also about 2.5B at large blurs (16, 32 min arc) but increased to more than 5B at the smallest blurs, where individual differences were also larger.

**Figure 6:**
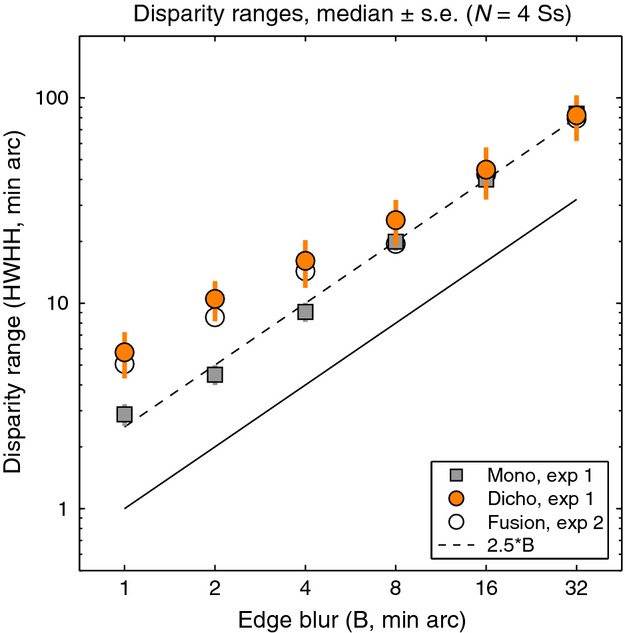
Experiment 1. Disparity ranges (median over the four observers) for single-central responses (derived as in *Figure 5*) plotted as a function of edge blur B (log-log plot). Squares: disparity range for the monoptic task was scale-invariant, at about 2.5B for all edge blurs. Coloured circles: for the dichoptic task, disparity range was similar to monoptic at large blurs, but increased to about 5.B at small blurs. For comparison, open circles show median estimates for the range of binocular fusion derived by modelling of experiment 2 (see text).

The group trends are summarised in *Figure 6* by the median σ_1_ ranges for monoptic and dichoptic viewing. The monoptic range (grey squares) represents the transition point between seeing a single edge and resolving two adjacent edges in the same (monocular) image. Interestingly, such scale-invariant resolution of two edges at an edge separation of 2.5B is exactly as predicted by a multiscale model of edge coding (*N*3+) described by Georgeson *et al*.^19^ (p.4 and their Figure S1). But we should bear in mind that this monoptic resolution might not be closely related to diplopia. The dichoptic task (filled circles in *Figure 6*) presumably involves binocular fusion, but how precisely its disparity range reflects the fusional range is unknown (see above). This is addressed more closely in experiment 2.

### Experiment 2: using modelling to derive disparity ranges for fusion and suppression

**Figure 7:**
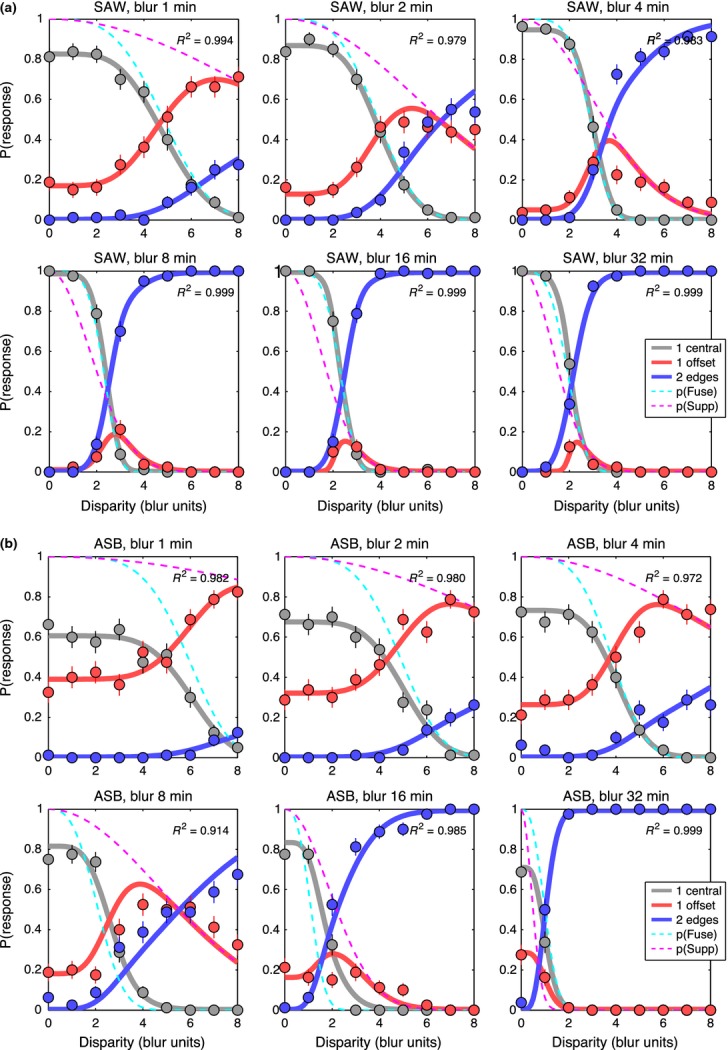
Experiment 2. Proportion of each response type in the 3-choice task is plotted as a function of disparity, in separate panels for each blur. Note how reports of 2 edges (diplopia, blue symbols) are much more frequent at larger disparities and larger blurs. (a) observer SAW. (b) observer ASB. Smooth solid curves show the fit of the model, with four free parameters for each panel (*c *=* *1). Dashed curves show model probabilities for the internal states of fusion *p*(*Fuse*) (cyan) and suppression *p*(*Supp*) (magenta). *R*^2^ is shown in each panel as a familiar guide to goodness-of-fit; all formal analyses were based on the deviance measure, more appropriate for these multinomial, frequency-count data.

For the three-choice task, the data are best seen by plotting all three response rates as a function of disparity. *Figure 7* shows results for two observers, chosen to illustrate both common trends and individual differences; plots for the other two are in the Supporting Information, Figure S1. Smooth curves show the model fits, which generally gave a good or excellent description of the data. As in experiment 1, ‘1 central’ responses fell with increasing disparity, but we now see that the ‘1 offset’ and ‘2 edges’ responses are quite distinct categories. Diplopia increased monotonically with disparity, and was much more likely at large blurs, while ‘1 offset’ responses behaved in a more complex fashion – rising with increasing disparity (at small blurs) or rising then falling (at larger blurs).

The model captures these varied patterns of data well, and parsimoniously (*Figure 7*). Responses ‘1 central’ are closely related to the model's underlying probability of fusion [*p*(*Fuse*), cyan dashed curve], but the rate of these ‘1 central’ responses (grey curve) often fell well below *p*(*Fuse*). This under-estimation is induced by positional noise, as already outlined in *Figure 3*, and is greater with narrower criteria (smaller *c*), or higher noise (*n*). In line with this analysis, observer ASB showed a large under-estimation effect (where the peak value of p(‘1 central’) fell between 0.6 and 0.8; *Figure 7b*) and for him the model returned consistently smaller *c/n* ratios than for the other observers across all conditions. For a 5-free fit, the median *c/n* ratio over the six blurs was 1.10 for ASB compared with SAW 2.32, DHB 2.70, SGB 1.49, and the outcome was similar for the 4-free and 3-free fits where *c* was fixed at 1.00. Thus the model gives us an important tool for separating task- and decision-related factors (noise and criterion) from sensory/perceptual factors that are specifically binocular (fusion and suppression).

**Figure 8:**
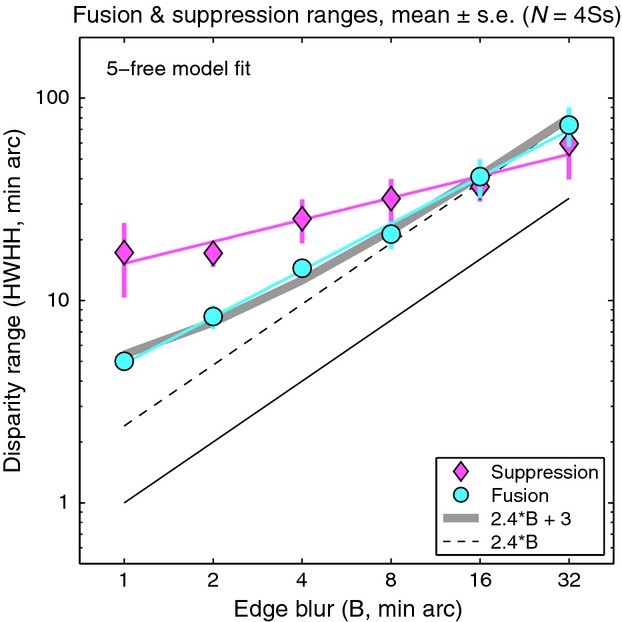
Experiment 2. Disparity range for fusion and suppression, estimated from model fitting (5-free), plotted as a function of blur on a log-log plot. Symbols show means of the four observers ± 1 S.E. Neither the fusion range (cyan line, slope 0.77) nor suppression range (magenta line, slope 0.36) is scale-invariant (dashed line, slope = 1), but the fusion range is closer to it. Grey curve represents a linear relation between fusion range and edge blur (fusion range = 2.4B + 3) – see Discussion.

**Figure 9:**
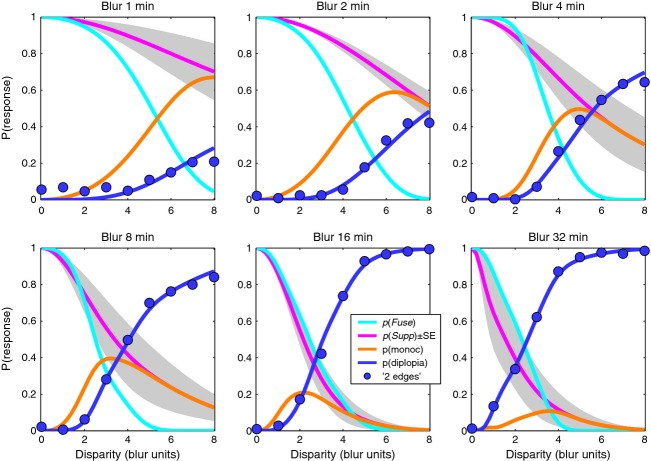
Experiment 2. Our interpretation of the results is summarized by model curves for probability of fusion *p*(*Fuse*) and interocular suppression *p*(*Supp*). Model curves were generated for each observer from fitted parameters (5-free fit), then group mean curves were plotted as shown. Shaded area represents ± 1 S.E. around *p*(*Supp*). Blue symbols show experimental group mean rates for the ‘2 edges’ response. Diplopia was higher at large blurs where suppression was restricted to smaller disparities.

Fusion and suppression ranges averaged over the four observers are plotted against edge blur in *Figure 8*. Mean fusional range was 2.5B at the larger blurs (B = 8, 16, 32 min arc) but increased to about 5.B at the smallest blurs. Disparity range for suppression was two to three times larger than the fusional range at small blurs, and this extensive range of suppression can account for the low rates of diplopia at small blurs. This is summarised more clearly in *Figure 9*, where smooth curves show fitted model probabilities, averaged over the four observers. By removing the perturbing effects of positional noise and criterion, these model curves should give us a more accurate view of fusion and suppression. The *monoc* state (orange curve) – corresponding to one visible edge and one suppressed edge – is most likely when *p*(*Supp*) (pink curve) is high and *p*(*Fuse*) (cyan curve) is low. This combination occurred most at small blurs. At larger blurs, on the other hand, the fusion and suppression ranges were about equal (*Figures 8, 9*). This led to high rates of diplopia, because suppression was absent at large disparities. Diplopia can occur only where suppression does not, and this combination was favoured at large blurs in our experiment. At small blurs suppression would presumably fall at even larger disparities (e.g. 10 – 20.B) and then diplopia would be seen more strongly, but at disparities beyond the range we tested.

In short, the rather complex, varied patterns of data across blurs and across individuals, seen in *Figure 7* and Figure S1, can be accounted for by two main factors: the range of suppression relative to that of fusion (σ_*s*_/σ_*f*_), and the spatial separation of the decision criteria relative to the positional noise (2*c/n*).

The average fusion and suppression ranges (*Figure 8*) were approximately power functions of blur with exponents (fitted slopes on the log-log plot) of 0.77 (fusion) and 0.36 (suppression). Both showed departures from scale invariance (a slope of one in this plot), but the fusion range was closer to scale invariance.

**Figure 10:**
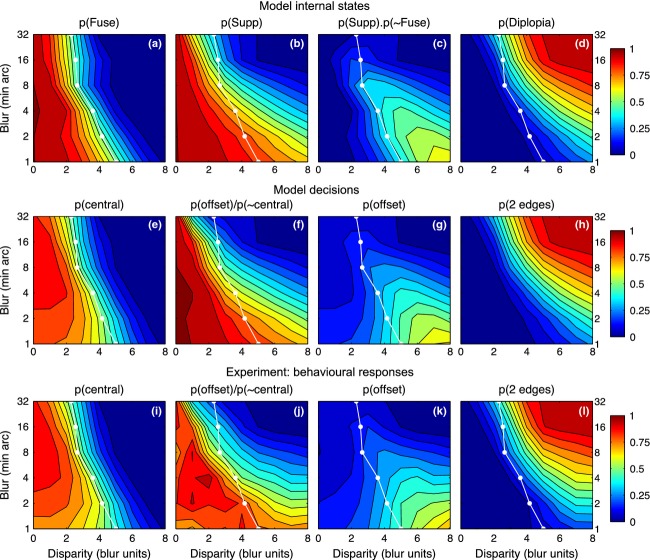
Experiment 2. Model internal states (top row, 5-free fit), model decisions (middle row) and experimental results (bottom row) summarised as surface contour maps. Horizontal axis represents disparity expressed as a multiple of the edge blur. Vertical axis plots edge blur B in min arc (log scale). White curve shows mean disparity range σ_*f*_ for fusion, derived from model fitting (map *a*). Surface colour codes probability of model states (a–d), model decisions (e–h) and mean proportion of responses over the four observers (i–l). Model states and decisions were derived by fitting to data separately for each observer, as in *Figure 7*; group mean maps are shown. Map *c* represents *p*(*monoc*) – the model's probability that there is no fusion and there is suppression of one monocular feature but not both. Maps *f* and *j* give an approximate estimate of interocular suppression derived directly from experimental data (j) or the corresponding model decisions (f) (see text for derivation), to be compared with the model map (*b*) for *p*(*Supp*). Note how double vision (d, h, l) is strongest at large blurs and large disparities, while the *monoc* state (c) and its experimental counterpart (the ‘1 offset’ response, *k*) are greatest at small blurs and large disparities.

The influences of disparity and blur, seen separately so far, can be visualised together as surface contour maps. The model maps (*Figure 10*, top row) show the group average probability of fusion (*a*), suppression (*b*) and diplopia (*d*) across the different levels of blur and disparity, inferred from model fits to data of all four observers. Panel *c* shows the model probability for seeing a *monoc* edge: the probability that interocular suppression occurs and fusion does not. It can be envisaged as (map *b*) × (1 − map *a*). With these maps it is easy to see how perceptual suppression of one edge occurs (at small blurs and large disparities, map *c*) only where the disparity range for suppression (*b*) extends well beyond the range for fusion (*a*). Diplopia (*d*) occurs beyond the fusion range, but only when the suppression range is small; this occurs at large blurs.

Maps *e, g, h* (middle row) show the model's ‘decisions’: that is, the model's account of the observer's behavioural responses (bottom row). There is clearly a good match between the two sets of maps. We emphasize that these are not predictions, because the model was fitted to the data for each subject and blur. Rather, the aim was to fit the model, incorporating the perturbing effects of criterion and noise, then remove those influences to derive the underlying characteristics of fusion and suppression, shown in the top row. Differences between maps in the middle and top rows (*e* vs *a*, *g* vs *c*) thus reflect the presence and absence of those influences respectively. The differences in these maps may not appear dramatic, so we computed difference maps (*e* minus *a*, *g* minus *c*, etc.) to examine this more closely (Figure S6). There was little difference between model decisions and observer responses (middle vs bottom rows of *Figure 10*) – confirming that the model fits the data well. But there was systematic structure (i.e. differences) between model states and model decisions (top vs middle rows of *Figure 10*), and this means that we are learning something new by removing the effects of noise. This is especially so for individuals like ASB, who adopted very narrow criteria for the ‘central’ judgement. For him, removal of these ‘crossover’ effects of noise (cf. *Figure 2*) was especially important, and made good sense of otherwise puzzling data.

On average, though, the observed response probabilities (*Figure 10; i,k,l*) are not a bad approximation to the internal states (*a*, *c*, *d*). This suggests an interesting simplification, in which we can derive an approximate map of suppression directly from the data, without model fitting. The reasoning is this: the observed *p*(‘central’) approximates the internal state *p*(*Fuse*)*,* while *p*(‘offset’) approximates *p*(monoc). Equation 3 tells us that *p*(monoc)* = p*(*Supp*).(1−p(*Fuse*)) so, replacing the model states with their approximations, we get:

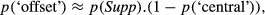

and therefore

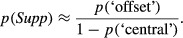
(7)

This ‘direct’ map of suppression is shown in *Figure 10j* (and for completeness the same treatment was applied to model decisions in *Figure 10f*). The similarity to the model-based suppression map (*Figure 10b*) is clear. All three suppression maps tell us that, perhaps counterintutively, interocular suppression between unfused monocular representations is greatest at small disparities and falls as disparity increases. This conclusion hinges on one assumption (Equation 3), but it is not the outcome of any other aspect of the modelling, because the empirical map of suppression (*Figure 10j*) shows it directly.

### Experiment 3

Our final experiment posed two further questions about the nature of fusion and suppression: (1) Does fusion occur for edges of opposite polarity? (2) How is the probability of fusion and suppression affected by relative contrast? We adopted the same model-based approach, but also included control trials whose purpose was to allow direct estimation of criterion and noise from the data.

**Figure 11:**
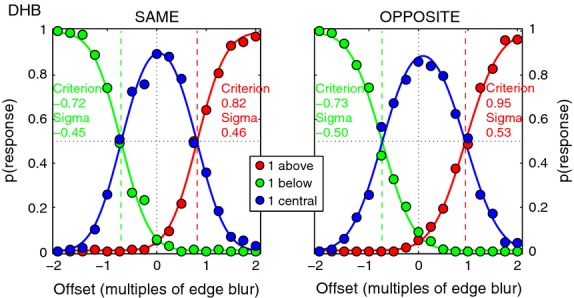
Experiment 3. Control data for one observer (DHB), averaged over the three contrast ratios. Blur B = 8 min arc. Edge pairs had zero disparity but their positional offset (same in both eyes) varied over trials from −2.B (below centre) to +2.B (above centre), with the same polarity (left) or opposite polarity (right). Plotting the proportion of position judgements (‘above’, ‘below’ or ‘central’) as a function of actual position allowed us to derive the position criteria (boundaries between the three position categories) and noise for each observer. A cumulative Gaussian (range 0–1) was fitted to the ‘below’ judgements (green) and the ‘above’ judgements (red). The 50% points on these curves gave the criterion positions, while noise was given by the S.D. (sigma) of the underlying Gaussian for each fit, as shown; see Table A2 for a full list, and Figure S5 for data of all four observers. The precision of these curve fits should make the parameter estimates very robust. Blue curve for ‘central’ judgements is not a fitted Gaussian, but is equal to 1 minus the sum of the other two curves (i.e. the three curves must sum to 1 at every offset position).

The experiment inter-mingled two kinds of trials: those with zero or non-zero disparity (similar to experiment 2), and the control trials that had zero-disparity but non-zero spatial offset. *Figure 11* shows typical results for these control trials, for one observer, and the legend describes how criterion and noise were derived from the fitted cumulative Gaussian curves. Similar plots for all four observers are shown in Figure S5, and Table A2 summarises the criterion and noise values derived. On average, the criterion boundaries were set about ±0.66B from the centre, and the noise standard deviation was about 0.5B, where B = 8 min arc.

**Figure 12:**
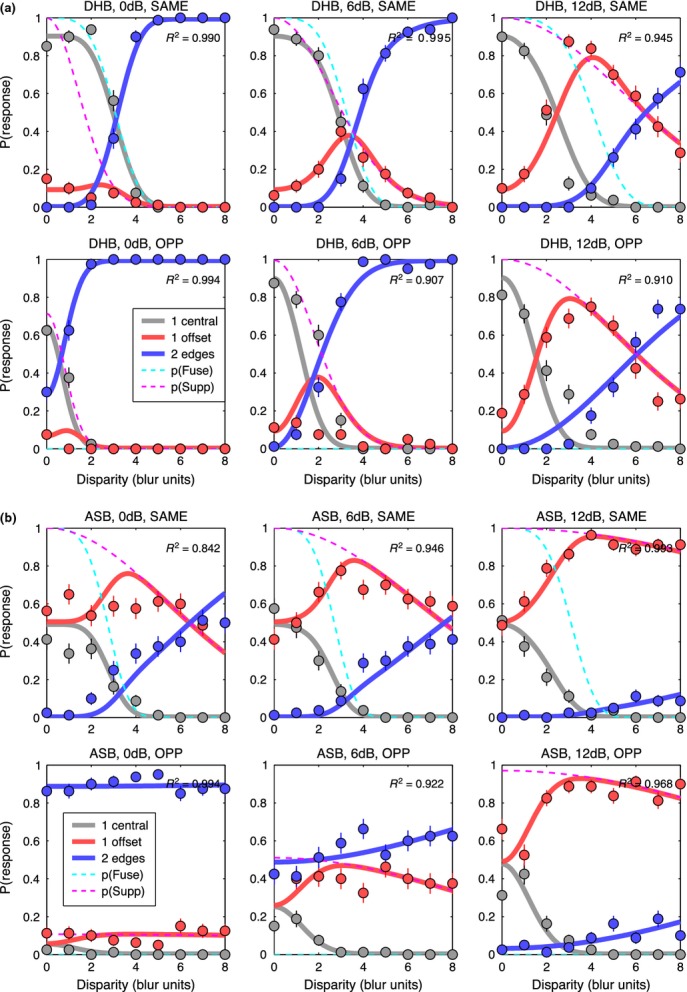
Experiment 3, edge blur 8 min arc only. Like *Figure 7*, proportion of each response type is plotted against disparity. Contrast imbalance (0, 6, 12 dB) increases across columns from left to right (interocular contrast ratios of 1, 2, 4). Note how reports of two edges (blue symbols) fell markedly when the two contrasts became unequal (second & third columns). (a) observer DHB. (b) observer ASB. Smooth solid curves show the fit of the model, assuming fusion for edges of the same polarity (top row in a, b), but not for opposite polarities (bottom row in a, b). Fitting had four free parameters; criterion and noise were fixed for each observer directly from experimental data (*Figure 11*). For disparities within the fusion range (about 0–3.B), diplopia was strong for opposite polarities, especially when contrasts were equal. This is consistent with an absence of fusion for opposite polarities.

For each observer, values of criterion and noise for the same polarity condition (Table A2) were used as fixed parameters in fitting the descriptive model to the main data, as described in *Model fitting* above, and illustrated in *Figure 12* for two observers (DHB, ASB; see Figure S2 for the other two). Individual differences in the data are quite marked, but there are also clear common features. For all four observers and for both same & opposite polarities, reports of diplopia (‘2 edges’) decreased with increasing contrast imbalance (left to right columns in *Figure 12*). Responses of ‘1-central’ were restricted to smaller disparities (below about 3.B) and were less frequent with opposite polarities.

Interestingly, if we took the position that all ‘1-central’ responses were based on fusion, we should conclude that fusion also occurred for opposite polarities, though less often than with same polarities (compare grey symbols in top and bottom rows of *Figure 12a* or *12b*). The modelling, however, shows that such a conclusion would be unsound. The model here (*Figure 12*) assumed no fusion for opposite polarities, and yet it describes the response rates and trends quite well. On this model, two edges of opposite polarity are superimposed in the binocular visual field, but even at small or zero disparity they don't fuse. The observer may report diplopia (at quite high rates, *Figure 12a, b*, bottom left panel), but if one of the two is suppressed, the observer reports a single edge. At small or zero disparity, the probability that this edge falls into the ‘central’ region can be quite high, as observed. Thus ‘1 central’ responses do not always mean fusion. Their frequency of occurrence in the opposite-polarity condition depends on disparity, criterion position and noise. These interactions are complicated and hard to think about, and the model serves as a useful tool for teasing them apart.

Using the model we compared four hypotheses, by allowing fusion to occur for (1) same polarity only, (2) both polarity conditions, (3) neither polarity condition, (4) opposite polarities only. In case (2) we constrained the fusion range to be the same for both polarity conditions. We considered model 1 to be *a priori* much more likely than the others; these model comparisons put that expectation to a formal test. In brief, model 1 (fusion only for same polarity) was uniquely favoured. This was confirmed firstly by detailed examination of the model-fitting errors (deviance residuals) which showed large, systematic errors for models 2,3,4, but comparatively little for model 1 (see Figure S4), and secondly by model comparison using AIC (Table B2). This shows that, of the four models considered, model 1 was uniquely the best (Akaike weight = 1 for each observer).

We also found that the equal-contrast (0 dB) condition was the most diagnostic one for distinguishing the models, in that the systematic difference in fitting errors between model 1 and the others was greatest there, and was least when the contrast difference was greatest (12 dB) (Figure S4). It seems intuitively reasonable that when one input is weak, models with and without fusion make similar predictions, but when the contrasts are equal they do not. Hence the equal-contrast condition discriminates best between the four models.

For both polarities, the probability of suppression always increased when the two eyes’ contrasts became more unequal (*Figure 12*, Figure S2). Examination of the original five-choice data (see Experiment 1), where observers had to identify whether a ‘1-offset’ edge was above or below centre, gave good information about which of the two edges was suppressed. When contrasts were unequal, it was always the lower-contrast edge that was suppressed.

**Figure 13:**
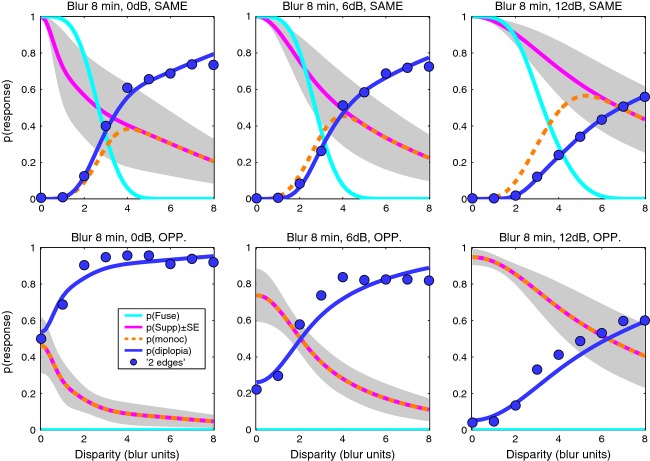
Experiment 3. Summary of results and model-based interpretation, in similar format to *Figure 9*. Top row: same polarity. Bottom row: opposite polarity. Symbols: group mean diplopia data. Left to right: Diplopia decreased as contrasts became more unequal, because suppression of one edge (the lower contrast edge) increased. Fusion (cyan curve) excludes diplopia at small disparities (0–2B, top row), but in the absence of fusion – with opposite polarities – diplopia can be quite strong even at small disparities (bottom left panel).

Group mean parameters for the best model are listed in Table A3. How these factors interact to produce perceptual outcomes is seen in *Figure 13* which summarises the findings of experiment 3, in the style of *Figure 9*, as averaged model curves for the same polarity (top row) and opposite polarity (bottom row). By looking at the model curves for ‘internal states’ we remove the perturbing influence of positional criterion and noise on the data. Despite some quite large individual differences in the degree of suppression and its spread across the disparity range, it seems reasonable to conclude from *Figure 13* – and the success of model 1 – that for the conditions of experiment 3 (B = 8 min blur, 200 ms duration):

1. Binocular fusion occurs for edges of the same polarity, for disparities up to a limit of about 2.5B to 3.B, as also found in Experiment 2.
2. Binocular fusion does not occur at all for edges of opposite polarity.
3. Interocular suppression falls monotonically with increasing disparity, for both same & opposite polarities, but
4. Interocular suppression expresses itself in perception only when fusion fails, either at larger disparities or with opposite polarities.
5. For opposite polarities: interocular suppression is relatively low when contrasts are equal, but increases when they become unequal. This occurs mainly by an increase in the peak probability, rather than increase in the disparity range.
6. For opposite polarities: fusion is absent, and so double vision is the complement of suppression, i.e. *p*(diplopia) = 1 − *p*(*Supp*) (*Figure 13*, bottom row).

## Discussion

We studied the characteristics of binocular fusion, using blurred edges with a range of vertical disparities. We chose vertical disparities to simplify the research question. For horizontal disparities there is abundant psychophysical and physiological evidence for a range of disparity-tuned mechanisms or ‘channels’,^25^,^26^ and so the fusional range, which is much larger for horizontal than for vertical disparities,^6^,^27^ probably reflects the total range that the set of disparity mechanisms can handle, rather than the disparity range of any one mechanism. It seems likely, though not certain, that multiple disparity ‘channels’ are absent for vertical disparities in central vision. Vertical disparities certainly carry information in peripheral vision, but there seems to be no sensation (and in particular no stereo depth) associated with vertical disparity in central vision. Thus by using vertical disparities, we aimed to tap into a simpler system where at a given spatial scale there might be just one ‘channel’, tuned to zero disparity.

### Interpreting perceptual decisions via descriptive modelling

We devised a simple probabilistic model to describe the way the internal states of binocular fusion and suppression lead to the observable, button-pressing responses. Given the underlying probabilities *p*(*Fuse*) and *p*(*Supp*), and the task-related parameters *c* and *n*, one can predict the probability of responses in the three-choice task. Our method put this into reverse, fitting the model to data in order to infer *p*(*Fuse*) and *p*(*Supp*). The model offers three benefits: the first is to remove the perturbing influence of positional noise, which was important for all observers at the small blurs, and for some observers (especially ASB) across a broader range of blurs. The second benefit, even when noise effects are small, is that one can deduce the underlying suppression function *p*(*Supp*), by combining two sets of response rates in a particular way (Equation. 7). This can also be done, to a fair approximation, directly from the data (*Figure 10j*).

The third benefit is that the model allows objective evaluation of different hypotheses about fusion phenomena. For example, we showed via the model that there is almost certainly no mechanism for perceptual fusion of edges with opposite polarity. This fits nicely with studies on stereo vision showing that depth from disparate edges is reliable only when the edges have the same polarity, i.e. the same sign of gradient, no matter whether the luminance levels forming the edge are similar or not.^28^ It is also consistent with evidence from binocular summation in contrast detection and contrast perception. The binocular advantage for detection of in-phase or same-polarity targets is lost – or reduced to the level of probability summation – when they are out of phase or of opposite polarity.^29^,^30^ Similarly, at low-contrasts, perceived contrast is enhanced for in-phase but not for out-of-phase gratings, which look similar in contrast to their monoptic counterparts.^31^ Polarity-specific neural summation,^32^ leading to fusion, is presumably a hard-wired strategy to combine matching image features that are likely to have arisen from the same source in the visual world.

### Individual differences

Individual differences in rates of diplopia, and in the inferred probabilities of suppression, were quite a feature of the data. These were subjective judgements, and so individual differences might reflect differences in response criteria, other than the positional criterion that we carefully accounted for. Devising suitable criterion-free tasks is one way of reducing such problems,^33^ though it is not straightforward to know what tasks are most suitable. Individual differences might also be a true reflection of the nature of interocular suppression at the neural level. In a strongly coupled, highly nonlinear network,^34^,^35^ small differences in network parameters between individuals could lead to large differences in the strength and dynamics of the suppressive effects.

### Scale invariance of fusion?

One of our key findings is that the disparity range for fusion increased markedly with blur (*Figure 8*). This confirms quantitatively the early demonstrations of Kulikowski.^4^ The blur-dependence of fusion, however, fell short of complete scale-invariance (the power exponent in *Figure 8* was 0.77 instead of 1.00). Despite the differences in stimuli and methods, these results show almost exactly the same trends as those reported for vertical disparity by Schor *et al*.,^5^ including the near-miss to scale invariance, and the increase in relative fusion range for sharp, high spatial frequency targets.

We considered whether blurring (in the monitor, the eye, or the visual system itself) might account for these departures from scale invariance. A small extra blurring would indeed affect small test blurs and not large ones, but this explanation still seems unlikely. We found that an assumed blur of about 1.5–2 min arc would predict the increased fusion range for B = 1, but it still under-predicted the fusion ranges at B = 2 or 4 min arc. More tellingly, if there were such a front-end blurring, we should expect to see a departure from scale invariance in the monoptic data as well (squares, *Figure 6*), but we see none. Its origin must be binocular. One candidate is vergence noise – the variation of ocular convergence over trials. But this too is likely to be small, about 1.5–2 min arc,^36^,^37^ and with such noise some trials would have a larger disparity than intended, but others would have a smaller one. Thus fusion would be less likely in the former, but more likely in the latter – so on average vergence noise should have little impact on the fusion curve. Similarly, the impact of any vergence constant error (vertical fixation disparity) should cancel out because we pooled data over both signs of disparity.

The near-miss to scale invariance would, however, be explained by a small vergence response to the disparity presented. A vergence response of (say) 3 min arc would reduce binocular disparity by that amount, and so increase the measured fusion range by the same amount. The sign of the vergence response would change with the sign of the stimulus disparity, and so its effect would not cancel out over trials. The grey curve in *Figure 8* thus represents the idea that the fusion range deviates from scale-invariance (at 2.4B, dashed line) only by an additive constant of 3 min arc. In short, the sensory fusion range might be truly scale-invariant, but slightly shifted by a small motor response. This response would have to occur quickly, because stimulus duration was only 200 ms. We have no direct evidence yet for our stimulus conditions, but the idea is at least plausible because short-latency changes in vergence of a few minutes of arc can be induced by both horizontal and vertical disparity within 80–100 ms.^38^ Any change in vergence would not affect monoptic judgements, and so these would remain scale-invariant, as observed in Experiment 1.

### Temporal factors

The influence of temporal factors on sensory fusion is also important in its own right. For horizontal disparity, the fusional range was much larger for slow modulations of disparity than fast modulations, but rate of change had much less influence on fusion for vertical disparity.^27^ This is consistent with the idea that the processing of depth from horizontal disparity is a slow, sluggish process.^39^–^41^ We used 200 ms presentation times, with vertical disparity. Would our estimates of fusion range have been much different at shorter or longer presentations? Probably not. In a supplementary experiment, one observer (author, SAW) collected data in the manner of experiment 2, for three blurs (2, 4, 16 min arc) at five durations (50, 100, 200, 400, 800 ms). Data and model fits are shown in Figure S7, with a summary of fusion and suppression ranges as a function of duration in Figure S8. The main result was that duration had only a minor effect on fusion range, at any blur. Disparity range for fusion increased by only 20–30% at 50 ms compared with 800 ms (Figure S8). This is fairly similar to earlier studies where fusion range was little affected by decreasing duration from 2000 to 160 ms, but modest (20–50%) increases were observed at a shorter duration (60 ms).^42^,^43^ Binocular combination and fusion may be rapid, while subsequent processing of stereo depth signals has more extended temporal integration, of at least 200 ms.^40^,^41^

### Single vision

Single vision occurs through fusion but also, beyond the fusion range, through suppression.^20^ *Figures 10a,c* map out these two kinds of single vision in some detail for the first time. From this perspective, single vision occurs on all those trials that are not diplopic – the light blue to dark blue regions of *Figure 10d*. On the relative scale of disparity, this full range of single vision is especially extensive for sharp edges (small blurs). This suggests that interocular suppression can serve as a mechanism for extending single vision to larger disparities. This extension can occur only where the suppression range exceeds the fusion range, and *Figure 10* shows that happens mainly for sharper edges. For those edges the fusion range is small in absolute terms (5–10 min arc), and so an extension of single vision (albeit without fusion) may be advantageous in natural viewing. But for large blurs the fusion range is already large, and so perhaps no such extension is needed and the suppression range can be relatively small, as observed. These blur-dependent characteristics of fusion and suppression should provide important constraints on the future modelling of fusion mechanisms in a multiscale visual system.

## APPENDIX

### Appendix A: Tables of parameters for Experiments 2 and 3

**Table A1:** Experiment 2: Group mean parameter values from four methods of estimation

	Blur B, min arc					
	1	2	4	8	16	32
Suppression range (B units)						
QAICc wtd mean	17.43	8.51	6.29	3.95	2.29	1.82
5-parameter fit	17.25	8.56	6.35	3.99	2.28	1.86
4-free, criterion = 1	17.23	8.36	6.17	3.91	2.29	1.79
3-free, criterion = 1, q = 4	17.25	8.37	6.16	3.91	2.30	1.80
Fusion steepness, q						
QAICc wtd mean	3.92	3.70	4.54	3.74	3.87	4.03
5-parameter fit	3.45	3.78	4.64	3.92	3.75	4.14
4-free, criterion = 1	3.40	3.62	4.13	3.85	3.65	3.85
3-free, criterion = 1, q = 4	4	4	4	4	4	4
Fusion range (B units)						
QAICc wtd mean	4.45	4.29	3.70	2.74	2.54	2.32
5-parameter fit	5.01	4.17	3.61	2.66	2.56	2.30
4-free, criterion = 1	5.04	4.56	3.87	2.92	2.46	2.34
3-free, criterion = 1, q = 4	5.34	4.74	3.90	3.02	2.55	2.32
Positional noise (B units)						
QAICc wtd mean	0.91	0.75	0.69	0.52	0.44	0.59
5-parameter fit	0.78	0.76	0.85	0.59	0.41	0.76
4-free, criterion = 1	0.78	0.68	0.59	0.46	0.46	0.53
3-free, criterion = 1, q = 4	0.81	0.68	0.60	0.50	0.48	0.56
Position criterion (B units)						
QAICc wtd mean	1.15	1.14	1.20	1.05	0.91	1.06
5-parameter fit	1.00	1.13	1.44	1.19	0.94	1.34
4-free, criterion = 1	1	1	1	1	1	1
3-free, criterion = 1, q = 4	1	1	1	1	1	1

**Table A2:** Experiment 3. Mean criterion and positional noise, in units of blur (B, 8 min arc), derived directly from the control data (*Figure 11*) without modelling

Polarity:	Same			Opposite		
Observer	criterion	noise	c/n ratio	criterion	noise	c/n ratio
SAW	0.84	0.26	3.21	0.82	0.34	2.43
DHB	0.77	0.46	1.69	0.84	0.52	1.63
ASB	0.38	0.57	0.66	0.35	0.73	0.48
RH	0.67	0.58	1.16	0.67	0.63	1.06
Group mean	0.66	0.46	1.68	0.67	0.55	1.40

**Table A3:** Experiment 3. Fitted & fixed parameters of the best-fitting model: group means

Parameter	Contrast ratio (dB)					
	0		6		12	
	Mean	S.E.	Mean	S.E.	Mean	S.E.
1. Fusion range (B units)	2.70	0.20	2.77	0.20	3.39	0.38
2. Fusion steepness, *q*	4		4		4	
3. Supp range SAME (B units)	4.14	1.77	4.89	1.49	9.06	3.37
4. Supp range OPP (B units)	8.29	5.72	5.38	2.20	8.09	3.12
5. Peak *p*(*Supp*) SAME	1		1		1	
6. Peak *p*(*Supp*) OPP	0.47	0.15	0.74	0.15	0.95	0.04
7. Posn. criterion (B units)	0.67	0.10	0.67	0.10	0.67	0.10
8. Posn. noise (B units)	0.46	0.07	0.46	0.07	0.46	0.07

Parameters 2 & 5 were fixed on the basis of experiment 2 and were the same for all observers. Parameters 7 & 8 were fixed in advance for each observer from experimental results, as shown in Table A2. The remaining four free parameters (1, 3, 4, 6) were estimated by optimising the model fit (maximum likelihood) separately for each observer and each contrast ratio.

## APPENDIX

### Appendix B: The Akaike Information Criterion (AIC) and multi-model inference

Our descriptive model was fitted to data of experiment 2 separately for each observer and each blur. With the five-parameter fit, there was some instability in the estimation of the noise and criterion parameters, because their ratio turns out to be more important than their absolute values (*Figure 4*), but errors in this estimation of noise and criterion might have induced biases in the parameters of greater interest – fusion range and suppression range. To stabilise the fitting, one can choose to fix one of the two correlated parameters (e.g. criterion) and then run a 4-parameter fit – but the choice of fixed parameter value is also uncertain. To overcome such problems of ‘model uncertainty’, contemporary modellers have used an approach called ‘multi-model inference’^44^ based on the Akaike Information Criterion (AIC).^45^ A very readable introduction to these methods is given by Symonds & Moussalli.^46^

In brief, several models are fitted and the AIC values for each model are computed from the error of the fit, taking into account the number of fitted parameters and the sample size. The relative plausibility of these *m* models is assessed by computing the set of Akaike weights *w* from the AIC values^47^. The weight *w*_*i*_ can be treated as the probability that the *i*th model is the best of the set. If *w*_*i*_ is close to 1 for one model (and therefore close to 0 for the others) then, of the models considered, that model is unambiguously best. But if the weights are more evenly distributed across the models, then no one model is best and each model carries some information about the true parameters. A better estimate of parameter *p* may be gained from ‘model averaging’: calculating the probability-weighted average of parameter values *p*_*i*_ across models: . To allow for the influence of sample size (number of data points contributing to the fit), one can begin by using a corrected expression AICc. To allow for additional noise in the data (‘over-dispersion’) – beyond that expected from binomial sampling or (in our three-choice method) trinomial sampling – one can use a modified AICc called QAICc.^46^ We used the method of maximum likelihood to fit the 4-parameter model to the data of experiment 2, with the criterion fixed at 0.5, 1, 1.5 or 2 times the blur B, then used the goodness-of-fit measure (deviance) to compute QAICc weights, and from these calculated the weighted average estimates of the parameters for each subject and blur. Individual values and group means for these parameters are shown in Table B1, and the group means are compared with the outcome of more conventional fitting in Table A1. The agreement between these group means is good, but the model-averaged QAICc estimates might well be more robust.^48^ These methods deserve closer attention in visual science.

**Table B1:** QAICc weighted average parameter values for each observer

	Blur B, min arc					
	1	2	4	8	16	32
Suppression range (B units)						
SAW	11.01	6.63	3.48	1.99	1.70	1.50
DHB	33.79	7.69	4.22	2.58	1.94	3.42
ASB	19.23	12.27	10.12	5.54	2.19	0.50
SGB	5.68	7.43	7.34	5.69	3.33	1.86
**Group Mean**	**17.43**	**8.51**	**6.29**	**3.95**	**2.29**	**1.82**
*S.E*.	*6.12*	*1.27*	*1.53*	*0.97*	*0.36*	*0.61*
Fusion steepness, q						
SAW	3.38	3.65	4.89	5.00	4.94	3.81
DHB	3.28	5.00	5.00	5.00	5.00	5.00
ASB	4.81	4.13	3.94	2.80	2.85	2.66
SGB	4.21	2.02	4.34	2.18	2.68	4.67
**Group Mean**	**3.92**	**3.70**	**4.54**	**3.74**	**3.87**	**4.03**
*S.E*.	*0.36*	*0.63*	*0.25*	*0.74*	*0.64*	*0.52*
Fusion range (B units)						
SAW	4.71	3.77	2.90	2.32	2.34	1.98
DHB	4.98	5.21	4.53	3.84	3.79	3.30
ASB	5.87	4.96	3.87	2.01	1.13	1.00
SGB	2.25	3.20	3.49	2.80	2.90	3.00
**Group Mean**	**4.45**	**4.29**	**3.70**	**2.74**	**2.54**	**2.32**
*S.E*.	*0.77*	*0.48*	*0.34*	*0.40*	*0.56*	*0.52*
Positional noise (B units)						
SAW	0.70	0.74	0.42	0.32	0.06	0.07
DHB	0.62	0.41	0.48	0.11	0.36	0.37
ASB	0.97	0.69	0.74	0.75	0.72	0.95
SGB	1.34	1.16	1.14	0.89	0.63	0.95
**Group Mean**	**0.91**	**0.75**	**0.69**	**0.52**	**0.44**	**0.59**
*S.E*.	*0.16*	*0.15*	*0.16*	*0.18*	*0.15*	*0.22*
Position criterion (B units)						
SAW	0.97	1.10	0.83	0.87	0.66	0.79
DHB	1.09	0.97	1.24	0.86	1.19	1.12
ASB	0.84	0.69	0.84	1.03	1.00	1.02
SGB	1.68	1.79	1.87	1.44	0.80	1.28
**Group Mean**	**1.15**	**1.14**	**1.20**	**1.05**	**0.91**	**1.06**
*S.E*.	*0.19*	*0.23*	*0.24*	*0.13*	*0.12*	*0.10*

**Table B2:** Experiment 3. Comparison of four models via the Akaike Information Criterion

Observer	Model	AICc	DiffAIC	Akaike wt
SAW	1	240.02	0.00	1
	2	623.34	383.32	0
	3	784.86	544.84	0
	4	859.54	619.52	0
DHB	1	432.81	0.00	1
	2	918.40	485.59	0
	3	1315.05	882.24	0
	4	1250.47	817.66	0
ASB	1	238.96	0.00	1
	2	1400.17	1161.21	0
	3	346.60	107.64	0
	4	1414.79	1175.83	0
RH	1	266.45	0.00	1
	2	1168.47	902.02	0
	3	510.97	244.53	0
	4	1218.33	951.88	0

To combine evidence for a given model over the three contrast ratios, we summed the deviance values for each of the three fits, then computed AICc accordingly, with a combined total of 12 free parameters over 81 data points. This was done separately for each observer. Model 1 (fusion only for same polarity) was uniquely favoured, for all four observers.

## Supporting Information

Figure S1 Experiment 2. As Fig. 7 of the main text, but showing results and model fits for the other two observers (DHB, above; SGB below). **Figure S2.** Experiment 3. As Fig. 12 of the main text, but showing results and model fits for the other two observers (SAW, above; RH below). **Figure S3.** Experiment 3. As Fig. 12 of the main text, but now the model included fusion of opposite polarities (with the same fusional disparity range as same polarities). **Figure S4.** Experiment 3. Goodness-of-fit assessed by residual error (deviance residuals). **Figure S5.** Experiment 3. A-D: Control data for the 4 observers. Blur B=8 min arc. **Figure S6.** Experiment 2. Difference maps discussed in the main text, in relation to the maps of Fig. 10. **Figure S7.** Supplementary experiment. The effects of stimulus duration on fusion, suppression and diplopia. **Figure S8.** Supplementary experiment, observer SAW. Summary of the effects of stimulus duration on fusion and suppression ranges, from data and model fits of Fig. S7.
