# Supplementary material for: Binocular fusion, suppression and diplopia for blurred edges
Source: Ophthalmic Physiol Opt. 2014 Jan 29;34(2):163–85. doi: 10.1111/opo.12108 (PMC4312971; doi:10.1111/opo.12108)
Supplement: Figure S1 — Experiment 2. As Fig. 7 of the main text, but showing results and model fits for the other two observers (DHB, above; SGB below). Figure S2. Experiment 3. As Fig. 12 of the main text, but showing results and model fits for the other two observers (SAW, above; RH below). Figure S3. Experiment 3. As Fig. 12 of the main text, but now the model included fusion of opposite polarities (with the same fusional disparity range as same polarities). Figure S4. Experiment 3. Goodness-of-fit assessed by residual error (deviance residuals). Figure S5. Experiment 3. A-D: Control data for the 4 observers. Blur B=8 min arc. Figure S6. Experiment 2. Difference maps discussed in the main text, in relation to the maps of Fig. 10. Figure S7. Supplementary experiment. The effects of stimulus duration on fusion, suppression and diplopia. Figure S8. Supplementary experiment, observer SAW. Summary of the effects of stimulus duration on fusion and suppression ranges, from data and model fits of Fig. S7. [file opo0034-0163-sd1.pdf]

Binocular fusion, suppression and diplopia for blurred edges

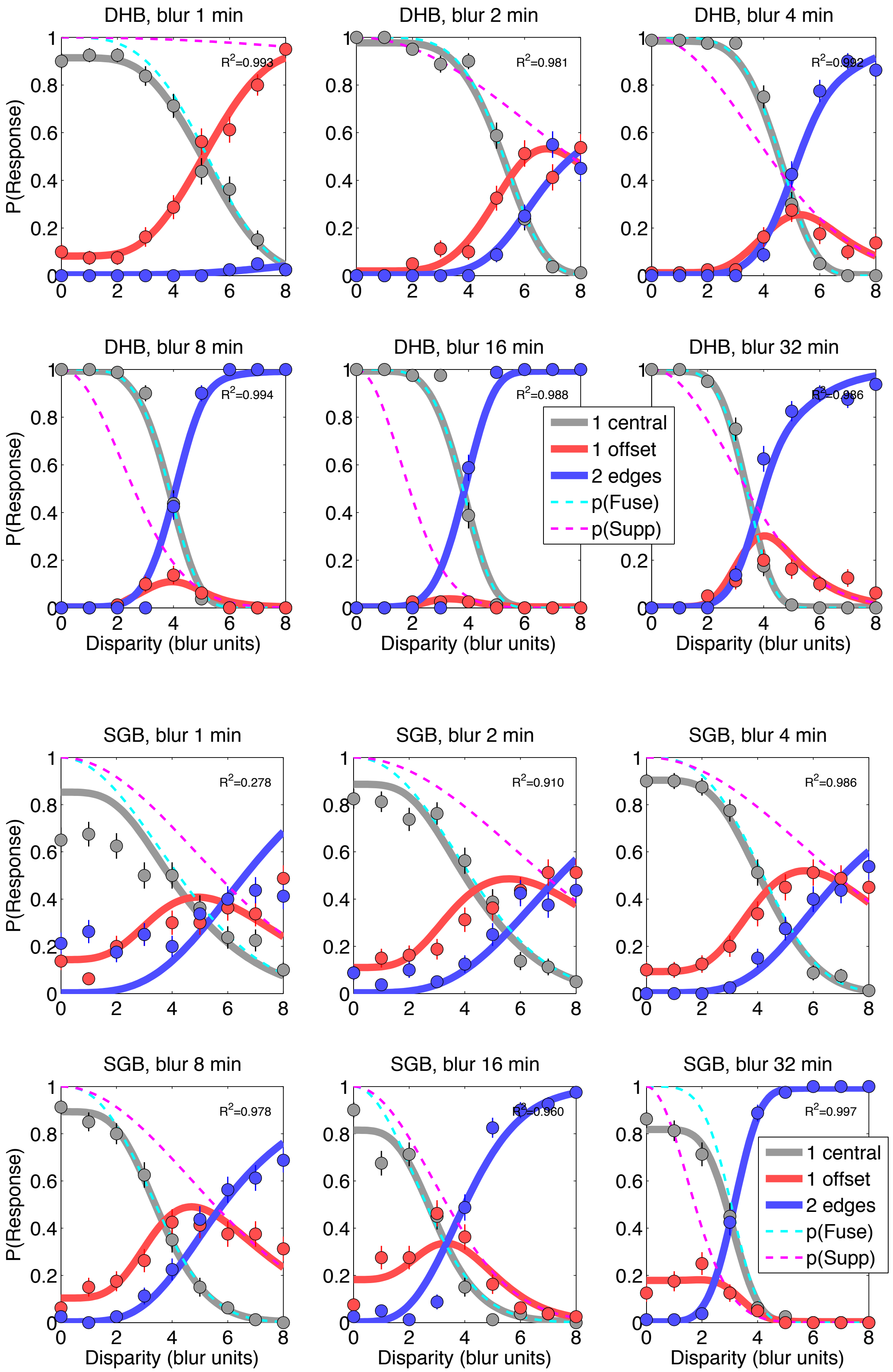

Figure S1. Experiment 2. As Fig. 7 of the main text, but showing results and model fits for the other two observers (DHB, above; SGB below)

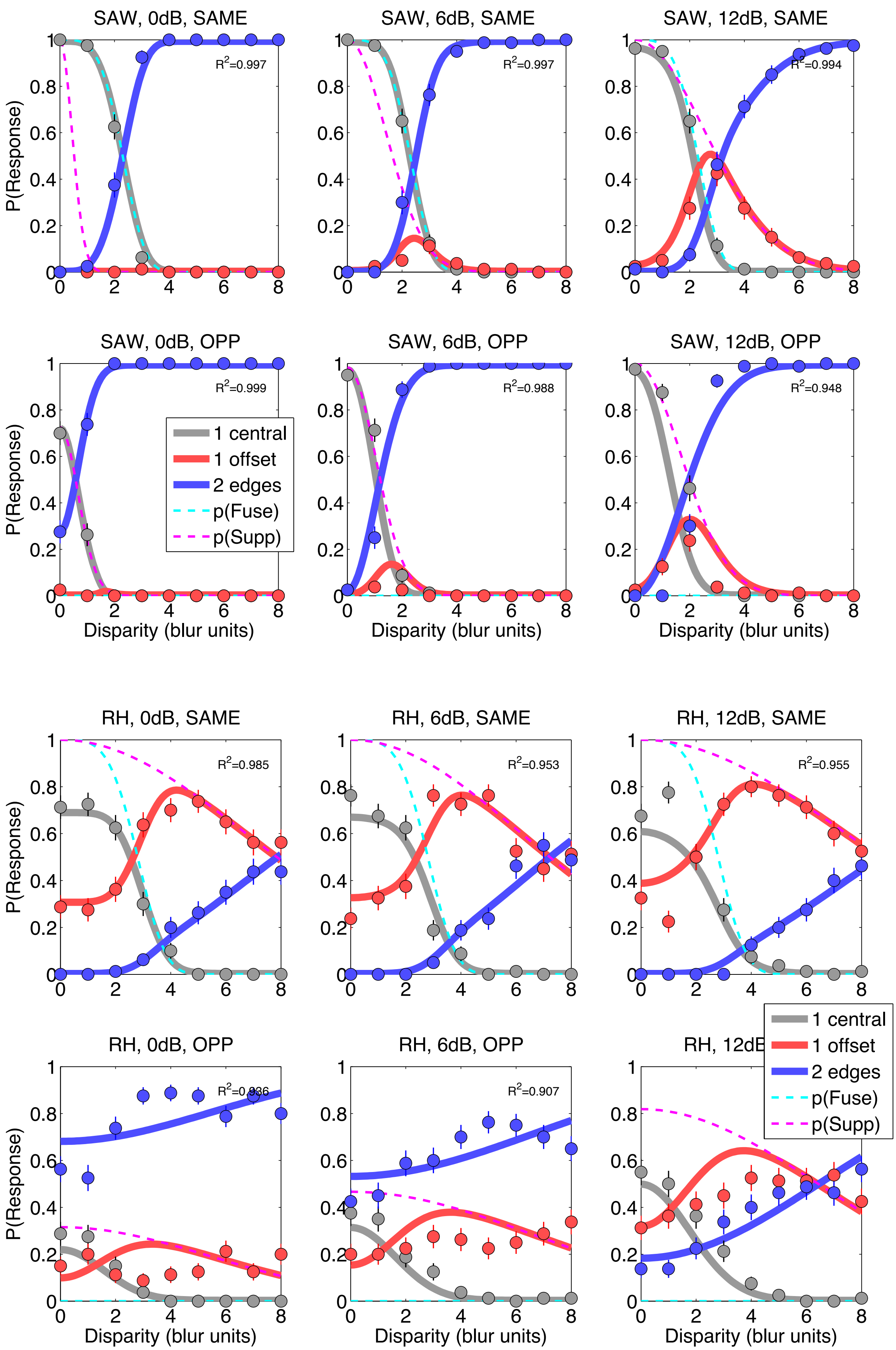

Figure S2. Experiment 3. As Fig. 12 of the main text, but showing results and model fits for the other two observers (SAW, above; RH below).

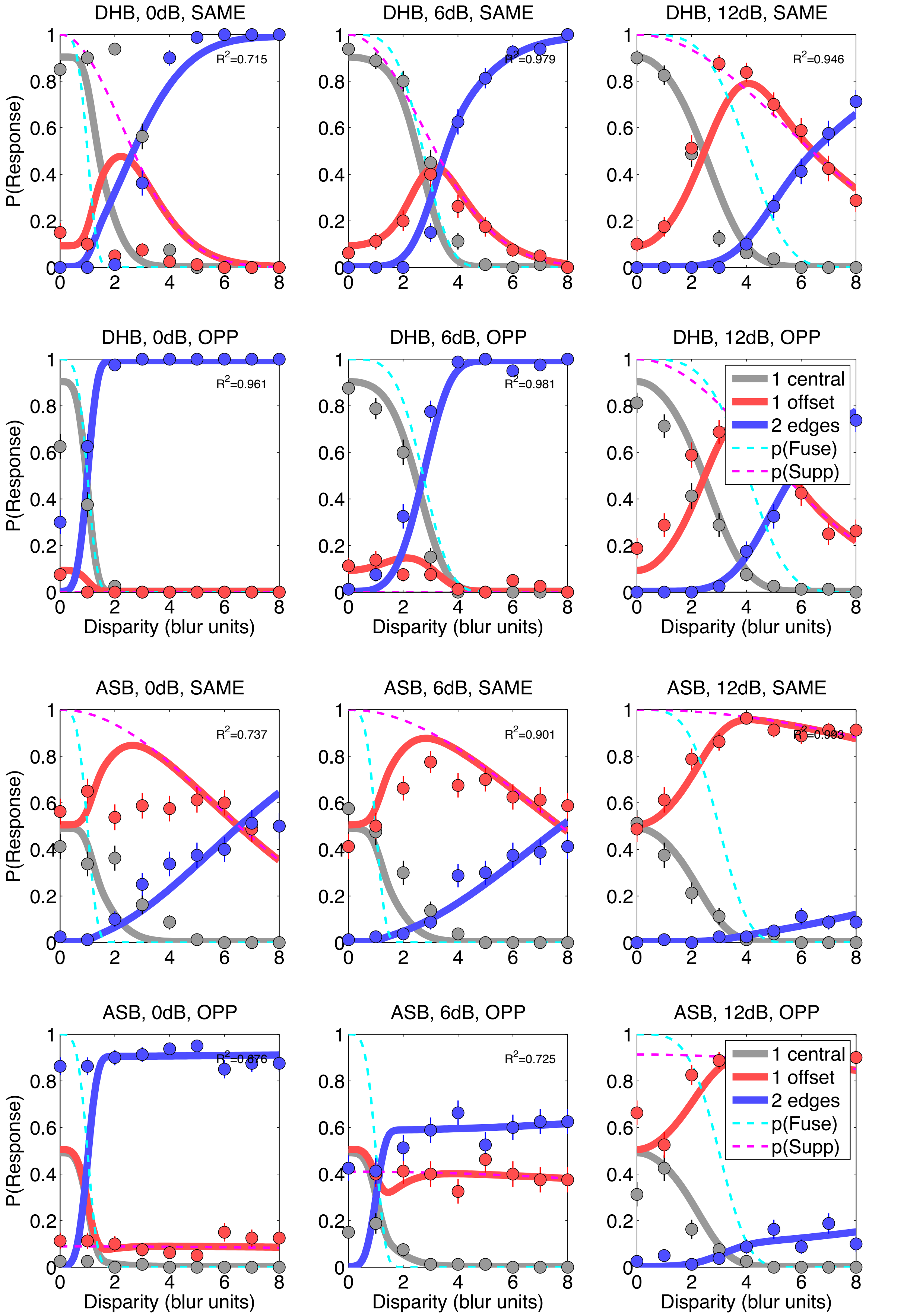

Figure S3. Experiment 3. As Fig. 12 of the main text, but now the model included fusion of opposite polarities (with the same fusional disparity range as same polarities). Observers DHB, ASB. Note the poor model fit for both same and opposite polarity at equal contrast (0dB). This confirms that binocular fusion of edge features of opposite polarity is very unlikely to occur. See also Fig. S4.

A. Model 1: Same polarities fuse, Opposites do not

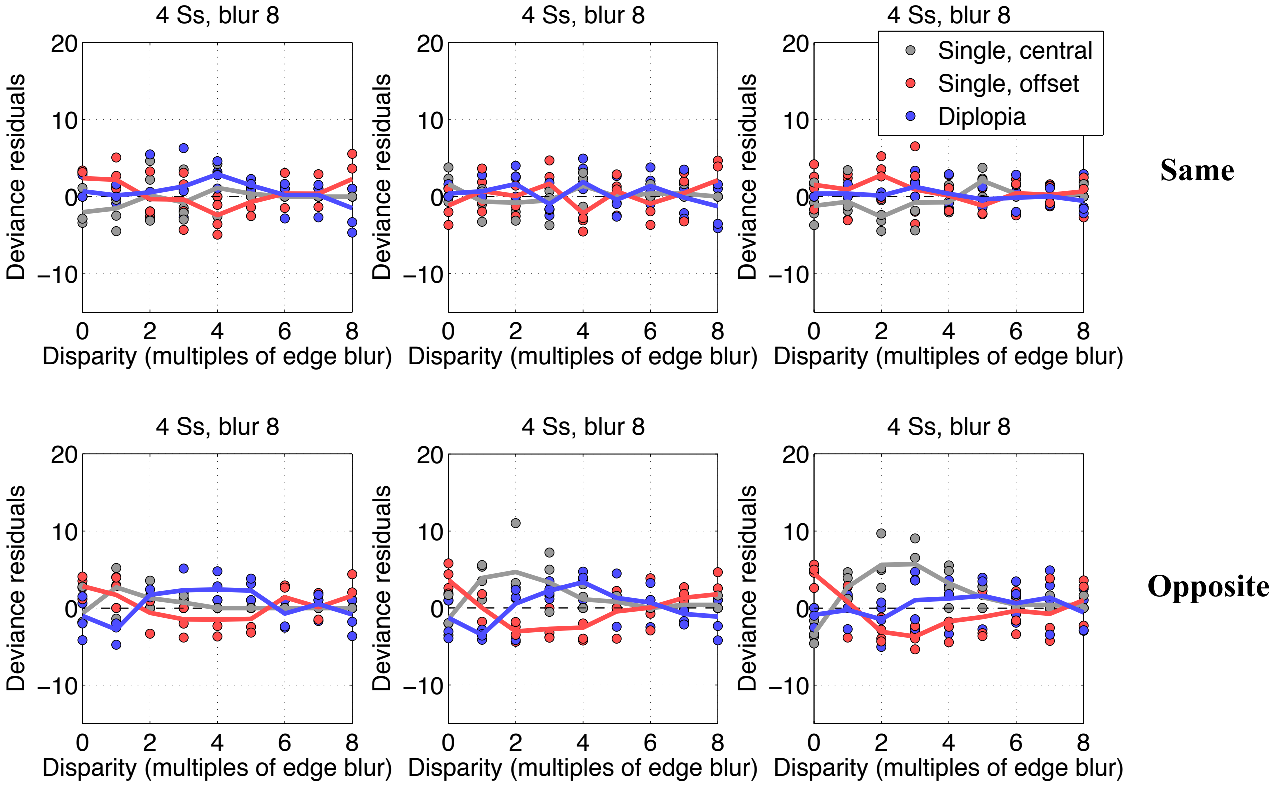

B. Model 2: Same polarities fuse & Opposite polarities fuse

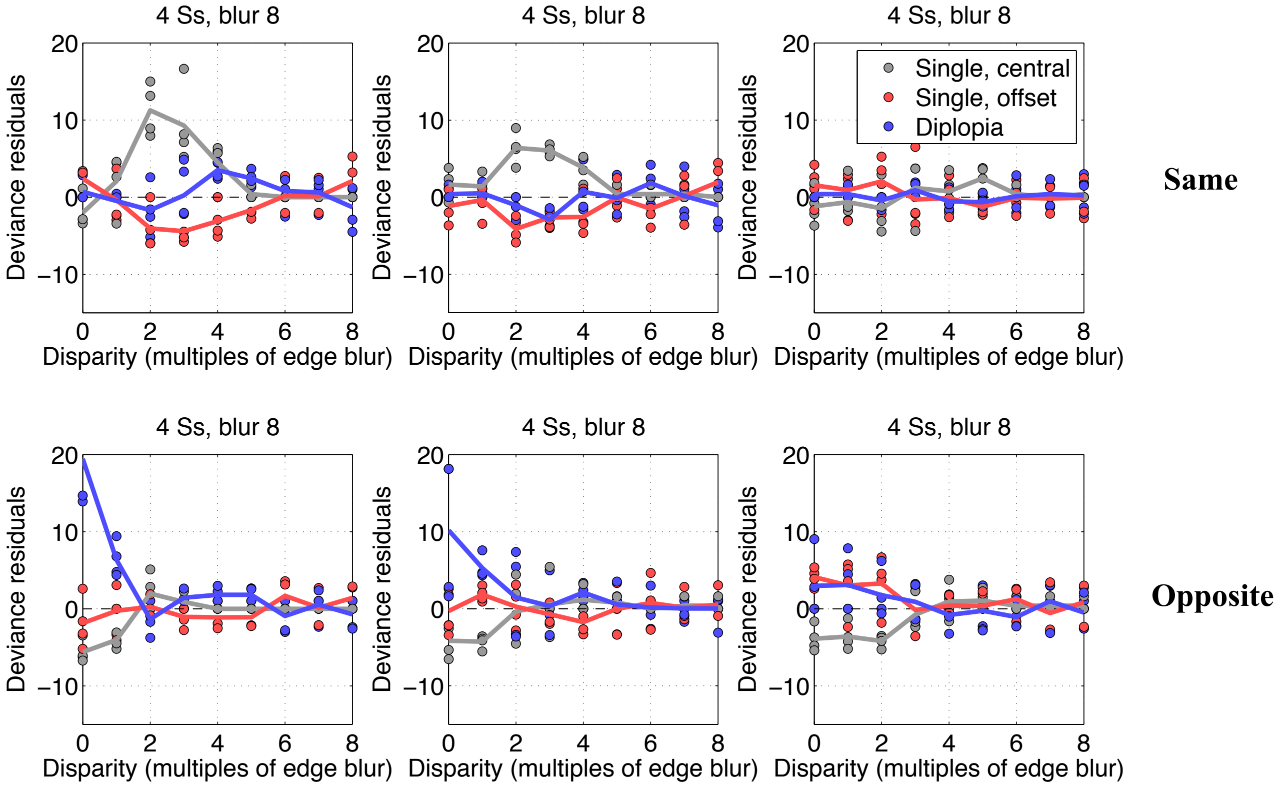

Figure S4. Experiment 3. Goodness-of-fit assessed by residual error (deviance residuals). A, B (above), C, D (below). For each model (A to D) top row is same polarity, bottom row is opposite polarity. *Legend continues below*

C. Model 3: No fusion at all

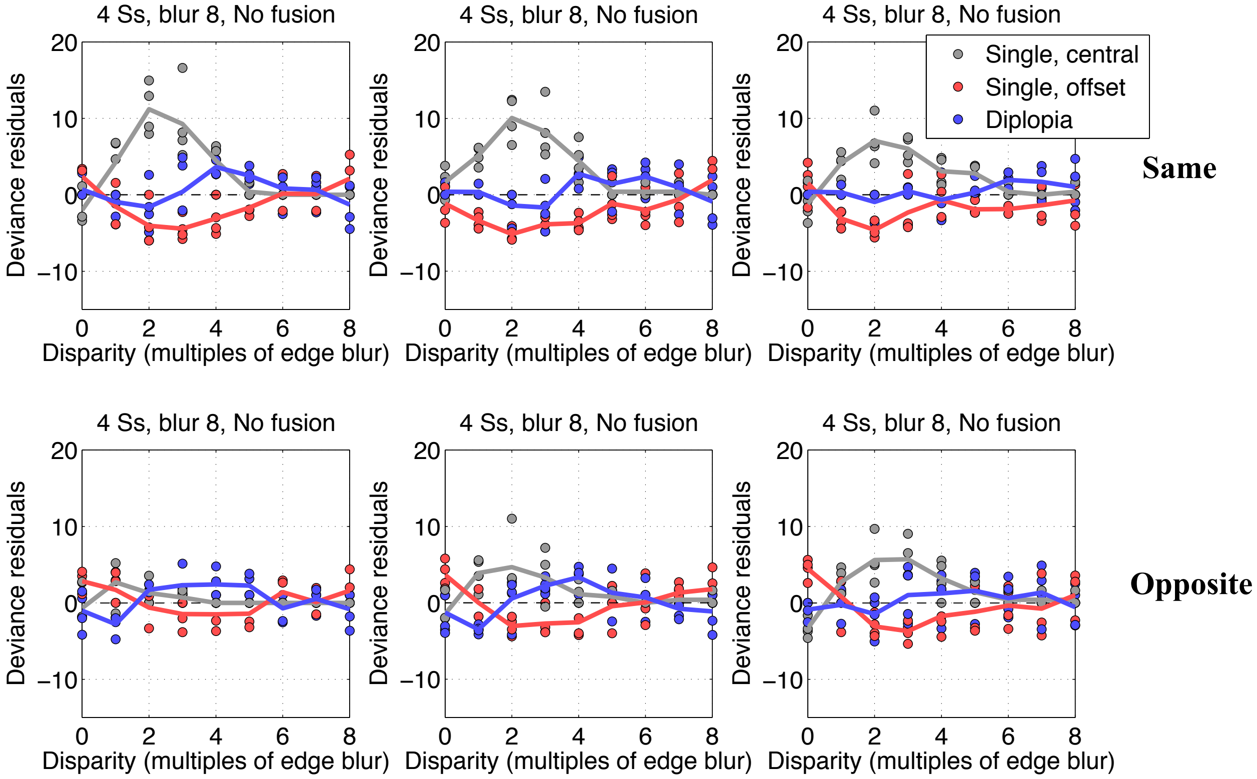

D. Model 4: Same polarities don't fuse, Opposites do

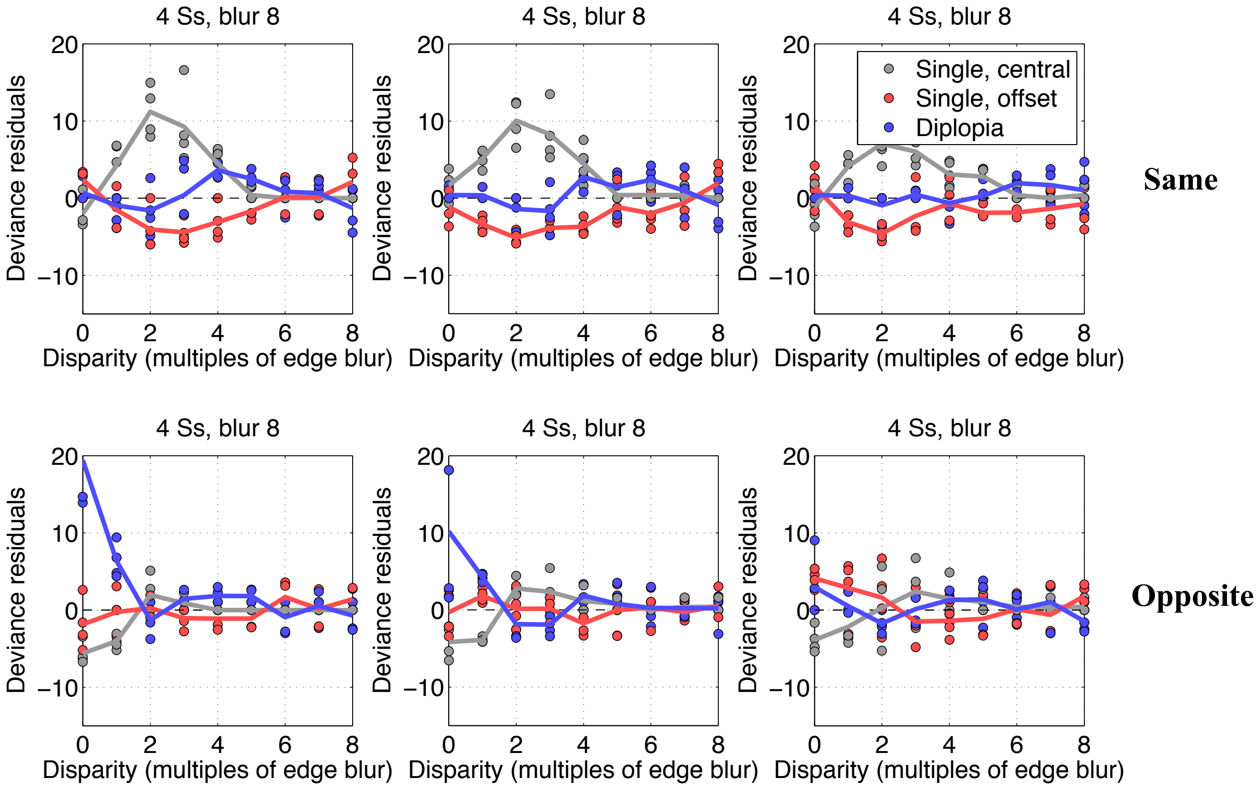

Figure S4. Experiment 3. Goodness-of-fit assessed by residual error (deviance residuals). Columns, left to right: contrast ratios of 1,2,4 (0, 6, 12 dB). A: Model 1 fused same polarities (top row) but not opposite polarities (bottom row). Overall, little or no residual structure indicates a good fit (cf. Figs. 12 and S2). Symbols, 4 observers; solid lines, group means. B: Model 2 assumed fusion of same & opposite polarities. C: Model 3 assumed no fusion at all. D: Model 4 assumed fusion only for opposite polarities. For models 2, 3, 4 strong residual structure for all observers (top and bottom rows) reflects systematic error of these models (cf. Fig. S3 for model 2). Failure of model 2 confirms that fusion is unlikely to occur for edges of opposite polarity.

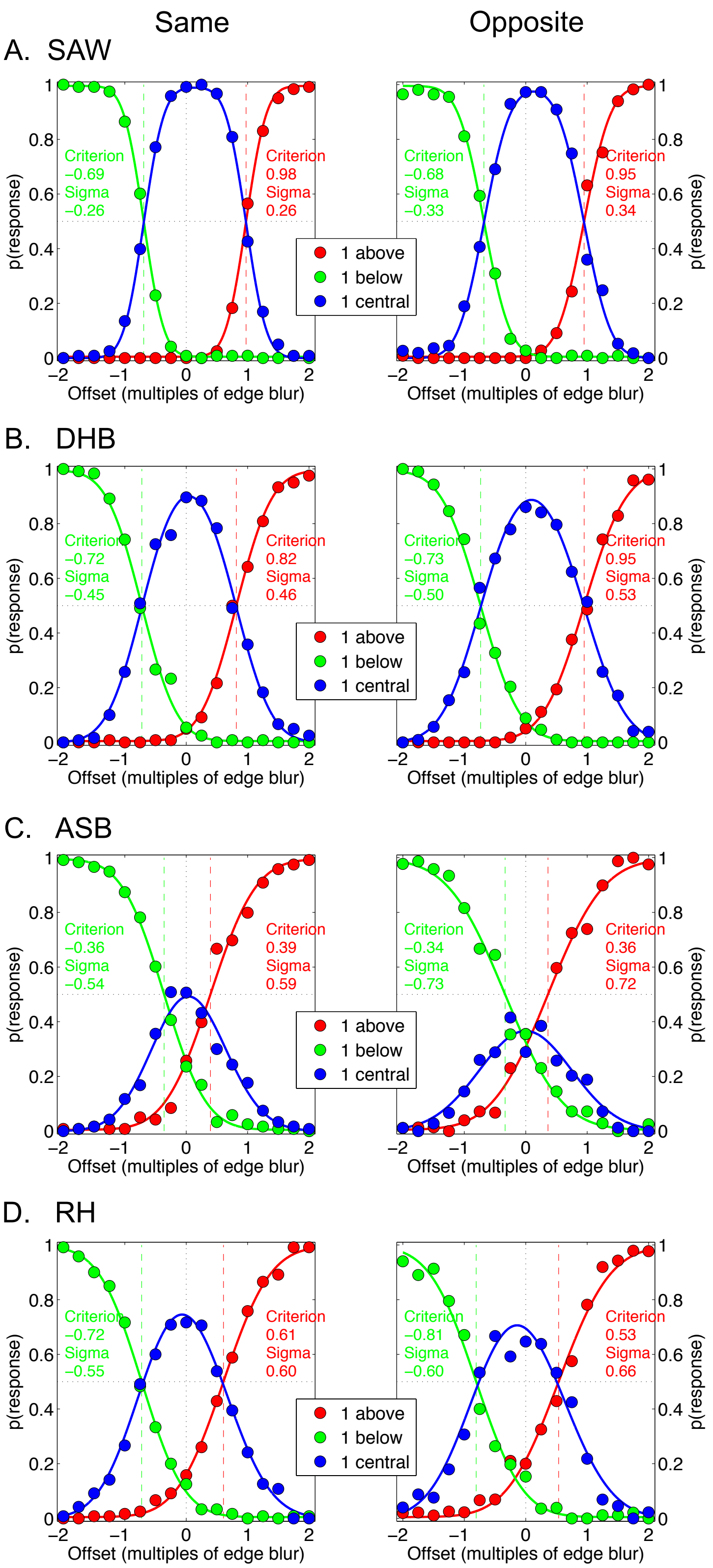

Figure S5. Experiment 3. A-D: Control data for the 4 observers. Blur B=8 min arc. Edge pairs had zero disparity but their position (same in both eyes) was offset over trials from -2.B (below centre) to +2.B (above centre), with the same polarity (left column) or opposite (right column). Plotting position judgements ('above', 'below' or 'central') as a function of actual position allowed us to derive the position criteria and noise for each observer. Cumulative Gaussian ogives (range 0 to 1) were fitted to the 'below' judgements (green) and the 'above' judgements (red). The 50% points on these curves gave us the criterion positions, while noise was the s.d. (sigma) of the underlying Gaussian for each fit, as shown. Blue curve for 'central' judgements is not a fitted Gaussian, but is equal to 1 minus the sum of the other two curves (ie. the 3 curves must sum to 1 at every offset position)

A. Experimental response rates minus Model response rates

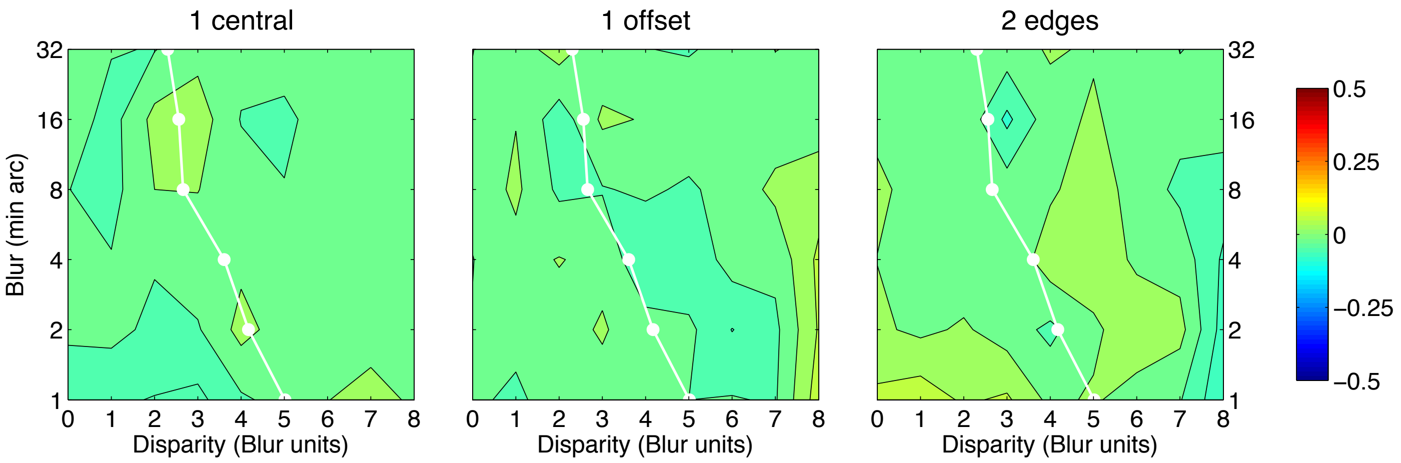

B. Model response rates minus Model state probabilities

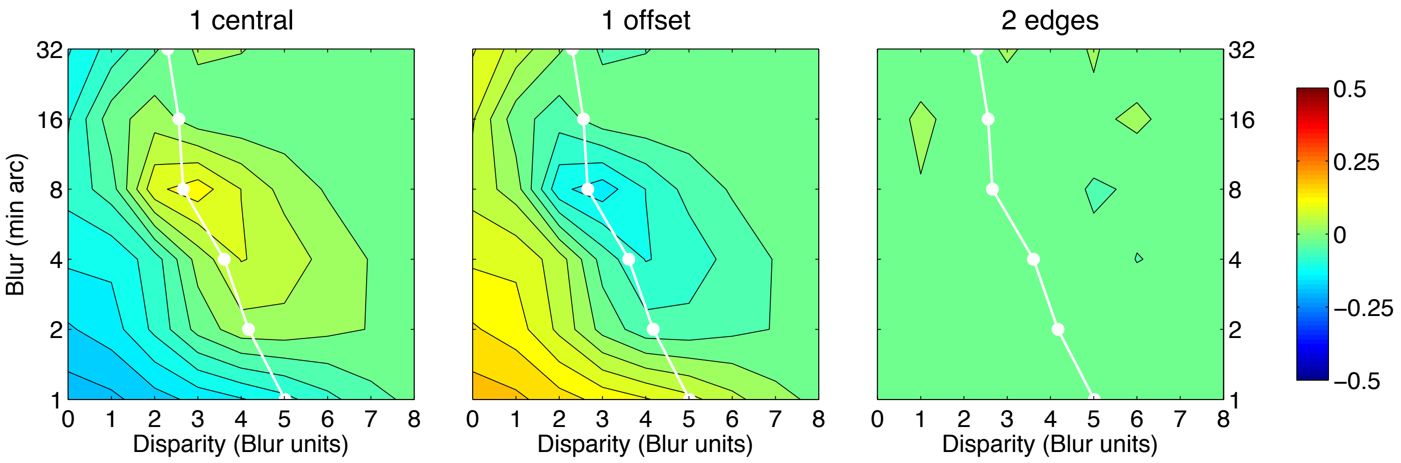

Figure S6. Experiment 2. Difference maps discussed in the main text, in relation to the maps of Fig. 10.
A: Experimental response rates minus Model response rates showed little systematic structure, reflecting a good fit of the model to the data.
B: Model responses minus Model state probabilities show clear structure, arising from the crossover of responses between 'central' and 'offset' categories induced by noise.

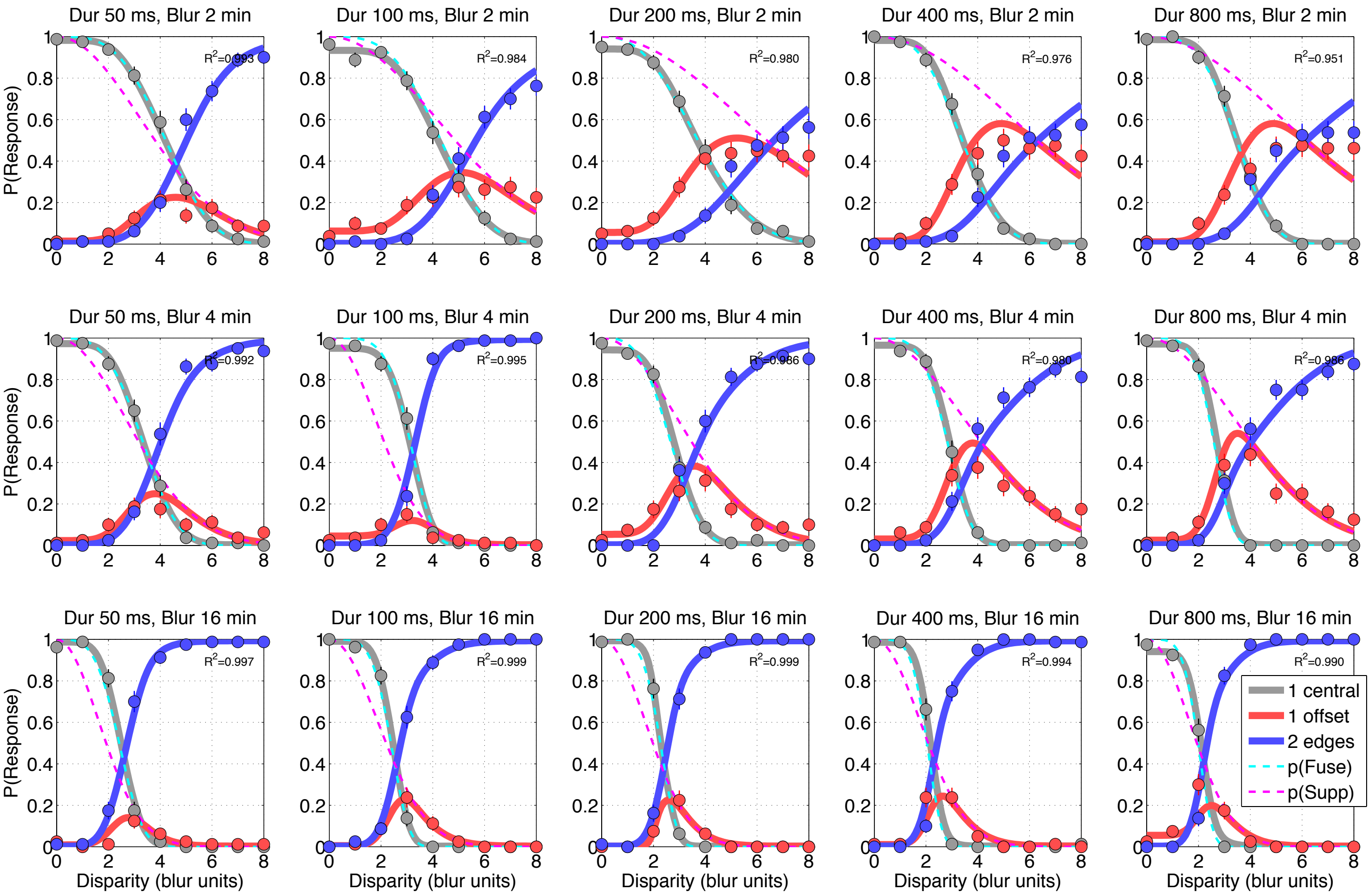

Figure S7. Supplementary experiment. The effects of stimulus duration on fusion, suppression and diplopia. Using the same experimental and modelling methods as experiment 2, one observer (author, SAW) was tested at 3 blurs (rows: B = 2, 4, 16 min arc) and 5 durations of presentation (columns: 50, 100, 200, 400, 800 msec). There were 4 free parameters (with fixed c=1) in the model fitting. Fusion range increased a little at short durations for all blurs, while suppression range increased (and diplopia decreased) at longer durations, but only for the sharper edges (B=2,4 min arc). Fig. S8 summarizes these effects more directly.

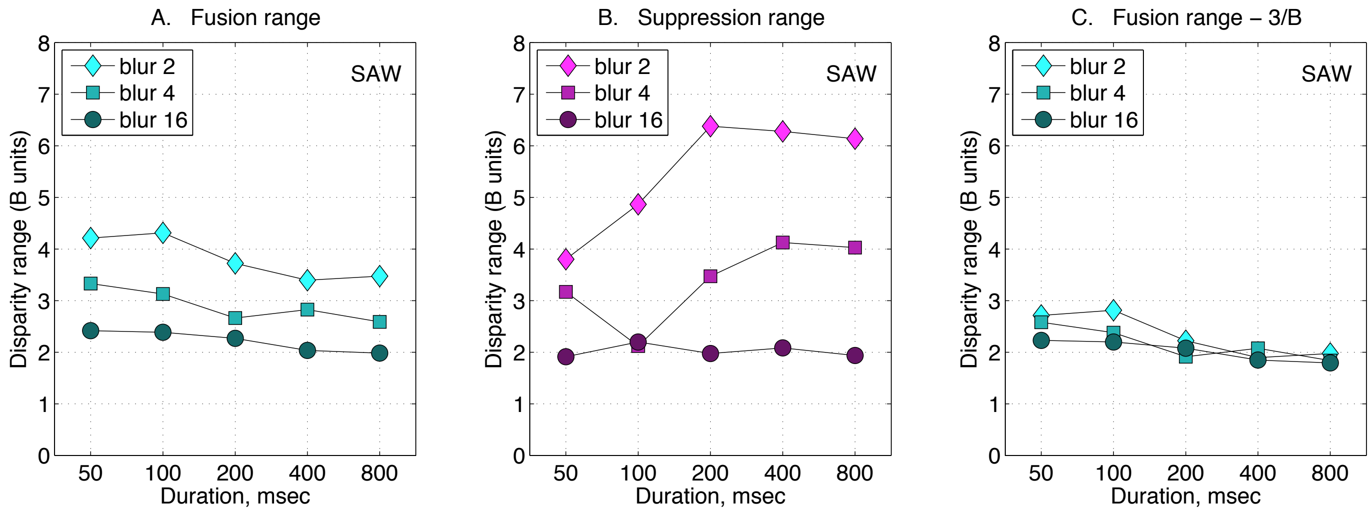

Figure S8. Supplementary experiment, observer SAW. Summary of the effects of stimulus duration on fusion and suppression ranges, from data and model fits of Fig. S7.

A: Fusion range, expressed in units of blur B, increased a little at short durations for all three blurs (mean increase 24% at 50ms *vs* 800ms). As in experiment 2, smaller blurs showed a larger fusion range.

B: Suppression range increased at longer durations, but only for the sharper edges (mainly at B=2 min arc).

C: Fusion range was almost independent of edge blur (i.e. was nearly scale-invariant) when a hypothetical additive shift of 3 min arc (3/B units of blur) was removed (subtracted) from the data of panel A. Such a shift (see main Discussion) could arise from a small vergence response to the presented disparity.
